# Color Stable Deep Blue Multi‐Resonance Organic Emitters with Narrow Emission and High Efficiency

**DOI:** 10.1002/advs.202302619

**Published:** 2023-07-09

**Authors:** Jihoon Kang, Soon Ok Jeon, Inkoo Kim, Ha Lim Lee, Junseop Lim, Jun Yeob Lee

**Affiliations:** ^1^ School of Chemical Engineering Sungkyunkwan University 2066, Seobu‐ro, Jangan‐gu Suwon Gyeonggi 16419 Republic of Korea; ^2^ Material Research Center, Samsung Advanced Institute of Technology Samsung Electronics Co., Ltd. 130 Samsung‐ro, Yeongtong‐gu Suwon Gyeonggi 16678 Republic of Korea; ^3^ Innovation Center Samsung Electronics Co., Ltd. Hwaseong Gyeonggi 18448 Republic of Korea; ^4^ SKKU Advanced Institute of Nano Technology Sungkyunkwan University 2066, Seobu‐ro, Jangan‐gu Suwon Gyeonggi 16419 Republic of Korea; ^5^ SKKU Institute of Energy Science and Technology Sungkyunkwan University 2066, Seobu‐ro, Jangan‐gu Suwon Gyeonggi 16419 Republic of Korea

**Keywords:** deep blue emitters, efficiency, narrow emission, spin‐vibronic coupling, thermally activated delayed fluorescence

## Abstract

The development of highly efficient and deep blue emitters satisfying the color specification of the commercial products has been a challenging hurdle in the organic light‐emitting diodes (OLEDs). Here, deep blue OLEDs with a narrow emission spectrum with good color stability and spin‐vibronic coupling assisted thermally activated delayed fluorescence are reported using a novel multi‐resonance (MR) emitter built on a pure organic‐based molecular platform of fused indolo[3,2,1‐*jk*]carbazole structure. Two emitters derived from 2,5,11,14‐tetrakis(1,1‐dimethylethyl)indolo[3,2,1‐*jk*]indolo[1′,2′,3′:1,7]indolo[3,2‐*b*]carbazole (tBisICz) core are synthesized as the MR type thermally activated delayed fluorescence emitters realizing a very narrow emission spectrum with a full‐width‐at‐half‐maximum (FWHM) of 16 nm with suppressed broadening at high doping concentration. The tBisICz core is substituted with a diphenylamine or 9‐phenylcarbazole blocking group to manage the intermolecular interaction for high efficiency and narrow emission. The deep blue OLEDs achieve high external quantum efficiency (EQE) of 24.9%, small FWHM of 19 nm, and deep blue color coordinate of (0.16, 0.04) with good color stability with increase in doping concentration. To the authors’ knowledge, the EQE in this work is one of the highest values reported for the deep blue OLEDs that achieve the BT.2020 standard.

## Graphical abstract

Deep blue organic light‐emitting diodes achieve high external quantum efficiency of 24.9%, small full‐width‐at‐half‐maximum of 19 nm, and deep blue color coordinate of (0.16, 0.04) with good color stability with increase in doping concentration.

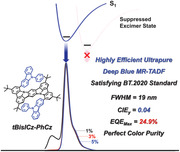

## 1 Introduction

High external quantum efficiency (EQE) and pure emission color are two key parameters of blue organic light‐emitting diodes (OLEDs) for commercial applications. In particular, the color purity is one of the most important specifications of blue OLEDs for universal use of them in various OLED products because it is closely related with the efficiency and device lifetime of the blue OLEDs.^[^ ^1^ ^]^

Several approaches have been pursued to realize the pure blue color with narrow emission spectrum.^[^ ^2^, ^3^, ^4^ ^]^ One of the most effective strategies is to build a multi‐resonance (MR) structure with an alternating highest occupied molecular orbital (HOMO) and lowest unoccupied molecular orbital (LUMO) within the chromophore.^[^ ^5^ ^]^ The MR structure is induced by electron rich and electron poor units embedded in the polycyclic aromatic hydrocarbon and allows thermally activated delayed fluorescence (TADF) by small singlet–triplet energy splitting (Δ*E*_ST_).^[^ ^6^, ^7^ ^]^ The most common method for the MR structure is to use boron as the electron deficient unit and sp^3^ nitrogen as the electron rich unit. The output of the boron and nitrogen‐based MR structure is high efficiency by large HOMO–LUMO overlap for radiative transition and narrow emission by the rigid backbone structure. Therefore, pure blue OLEDs with high EQE can be afforded as exemplified in several works reporting DABNA‐1, *ν*‐DABNA, and so on.^[^ ^5^, ^8^, ^9^, ^10^ ^]^ Other than the boron‐nitrogen framework, boron‐oxygen, boron‐sulfur, and carbonyl‐nitrogen scaffolds were demonstrated as the MR core structures.^[^ ^11^, ^12^, ^13^, ^14^ ^]^ However, the boron‐nitrogen‐based MR‐TADF compounds cannot fulfill the BT.2020 color specification requiring *y* color coordinate of below 0.05 despite sharp emission spectrum and high EQE, while other MR derivatives cannot satisfy the narrow emission spectrum with full‐width‐at‐half‐maximum (FWHM) less than 30 nm. Although our group reported pure carbon and nitrogen‐based MR‐TADF emitters with *y* color coordinate of 0.05, the emission spectrum was largely broadened by strong intermolecular interaction. Therefore, the MR‐TADF emitters satisfying the small FWHM, *y* color coordinate under 0.05 with good color stability, and high EQE are essential.

Here, we describe deep blue MR‐TADF compounds emitting at a peak wavelength of 446 nm with a small FWHM of 19 nm and doping concentration resistant emission spectrum. A molecular framework of 2,5,11,14‐tetrakis(1,1‐dimethylethyl)indolo[3,2,1‐*jk*]indolo[1′,2′,3′:1,7]indolo[3,2‐*b*]carbazole (tBisICz) was the MR‐TADF core up‐converting triplet excitons by spin‐vibronic coupling (SVC) assisted reverse intersystem crossing (RISC).^[^ ^15^, ^16^ ^]^ It was modified with diphenylamine (DPA) or 9‐phenylcarbazole (PhCz) to manage the SVC process and intermolecular interaction. It was demonstrated that the tBisICz derivatives achieved color coordinate of (0.16, 0.04), small FWHM of 19 nm with little broadening at high doping concentration, high EQE of 24.9%, and little concentration quenching effect. The device data established in this work are the state‐of‐the‐art performances of the deep blue OLEDs in terms of EQE and color coordinate.

## 2 Results and Discussions

### 2.1 Design and Synthesis

The tBisICz core is a modified version of 2,5,13,16‐tetra‐*tert*‐butylindolo[3,2,1‐*jk*]‐indolo[1′,2′,3′:1,7]indolo[2,3‐*b*]carbazole (tDIDCz) which was reported as a narrow‐emitting core with pure violet emission.^[^ ^17^ ^]^ The *para* orientation of two nitrogens in the tBisICz core shifted the emission color from violet of tDIDCz to pure deep blue, enabling the application of tBisICz as a pure deep blue emitting chromophore. Additionally, the fusion of the two indolo[3,2,1‐*jk*]carbazole (ICz) units rigidifies the molecular structure for narrow emission and delivers TADF characteristics despite of rather large Δ*E*_ST_ by SVC mechanism. Therefore, the tBisICz is appropriate as the core structure of pure deep blue emitter, but it suffers from strong intermolecular interaction due to planar molecular structure and relatively low photoluminescence quantum yield (PLQY).^[^ ^18^ ^]^ The issue can be circumvented by employing a PLQY enhancing and SVC facilitating blocking groups in the tBisICz core structure without disrupting the MR properties.

**Figure 1:**
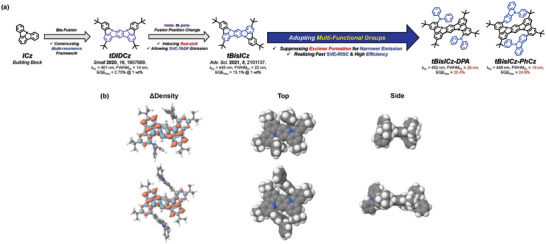
a) The design concept of ICz derived MR‐TADF emitters. b) Difference density plot upon S_0_–S_1_ excitation with the decreased/increased electron density in red/blue. Top and side views of a van der Waals sphere representation.

We selected DPA and PhCz, which were introduced symmetrically at the *ortho*‐position of N atoms of tBisICz core to control intermolecular interaction by steric hindrance, providing 2,5,11,14‐tetrakis(1,1‐dimethylethyl)‐*N* ^7^, *N* ^7^, *N* ^16^, *N* ^16^‐tetraphenyl‐indolo[3,2,1‐*jk*]‐indolo[1′,2′,3′:1,7]indolo[3,2‐*b*]carbazol‐7,16‐amine (tBisICz‐DPA) and 2,5,11,14‐tetrakis(1,1‐dimethylethyl)‐7,16‐bis(9‐phenyl‐carbazol‐3yl)‐indolo[3,2,1‐*jk*]‐indolo[1′,2′,3′:1,7]indolo[3,2‐*b*]carbazole (tBisICz‐PhCz) (**Figure** **1**a).

The structurally shielded two MR‐TADF emitters, tBisICz‐DPA and tBisICz‐PhCz, were prepared by following the synthetic method in Schemes S1 and S2, Supporting Information. Similar synthetic method substituting the multi‐functional blocking groups followed by coupling and fusion was adopted in the two emitters. Detailed description of the synthesis process is provided in Experimental section of Supporting Information.

First‐principles calculations were performed to understand the effect of shielding groups on the photophysical transitions of the MR‐TADF emitters. Figure 1b shows the difference density plot of tBisICz‐DPA and tBisICz‐PhCz upon the S_0_–S_1_ excitation at the SCS‐ADC(2)/def2‐SVP level of theory. The employed method incorporates higher‐order electron correlations compared to the single‐excitation methods such as TDDFT, providing a balanced account for the S_1_ and the T_1_ energies, and hence assuring an accurate Δ*E*_ST_.^[^ ^19^, ^20^ ^]^ The multiple‐resonance alignment is also well displayed as the alternating distribution of the increased and decreased density within the fused aromatic plane even after substitution of the blocking groups. We calculated the adiabatic singlet‐triplet gap (Δ*E*_ST_) and obtained 0.24 and 0.26 eV for tBisICz‐DPA and tBisICz‐PhCz, respectively, which are remarkably similar to that of tBisICz (0.26 eV). With the retained MR character, this demonstrates that our meticulous substitutions at the 1‐ and 5‐positions have little effect on the excited‐states, while effectively providing a shielding from the inter‐molecular interactions.

To quantitatively assess our design principle, we calculated the RISC rates (*k*_RISC_) of the tBisICz derivatives by analytically solving the Fermi golden rule rate equation with the spin‐vibronic Hamiltonian,^[^ ^21^, ^22^ ^]^ which treats the spin‐orbit and the nonadiabatic couplings on equal footing to the second‐order. We obtained *k*_RISC_ of 168 s^−1^ for tBisICz‐DPA and 41 s^−1^ for tBisICz‐PhCz at 300 K; these values are in the similar time scale with the theoretical value of the base tBisICz (150 s^−1^) obtained at the same level of theory,^[^ ^15^ ^]^ implying noticeable TADF activity. More importantly, the spin‐vibronic contribution in *k*_RISC_ for both emitters are ≥95% (Table S1, Supporting Information), reaffirming that these molecules behave as SVC‐TADF emitters as the original tBisICz where the triplet‐to‐singlet transition is largely afforded by the energy‐resonance between the S_1_ and the T_2_ states.

### 2.2 Photophysical Characterization

**Figure 2:**
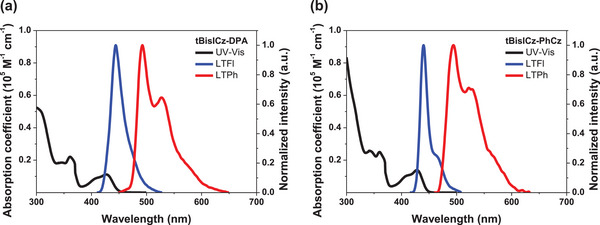
The UV–vis absorption and normalized PL spectra of a) tBisICz‐DPA and b) tBisICz‐PhCz.

Photophysical properties of the MR‐TADF emitters were measured and summarized in **Table** **1**. The ultraviolet–visible (UV–vis) absorption spectral pattern from dilute tetrahydrofuran solution (1.0 × 10^−5^ m) was analogous in the two emitters as shown in **Figure** **2**, but the absorption coefficient was large in the tBisICz‐PhCz due to intense absorption by local electronic transition of the tBisICz core. In the case of tBisICz‐DPA, weak CT nature decreased the UV–vis absorption. The photoluminescence (PL) spectra from dilute tetrahydrofuran solution collected at 77 K reflected the CT and local transition nature of the two emitters. A weakly widened PL spectrum with FWHM of 27 nm was delivered from the tBisICz‐DPA emitter, whereas a very sharp spectrum with FWHM of 16 nm was served from the tBisICz‐PhCz emitter. Moreover, the peak wavelength was red‐shifted by 4 nm in the tBisICz‐DPA emitter. These results can be interpreted as the effect of weak CT character of tBisICz‐DPA due to the strongly electron‐donating DPA blocking group.^[^ ^23^ ^]^ The singlet energy (*E*_S_) was reduced from 2.82 to 2.79 eV by the DPA substitution, which enlarged the Stokes shift of tBisICz‐DPA to 17 nm compared with 12 nm of tBisICz‐PhCz. The phosphorescence spectra were similar, affording triplet energy (*E*_T_) of 2.51 eV in the two emitters. The small singlet energy decreased the Δ*E*_ST_ of tBisICz‐DPA up to 0.28 eV relative to 0.31 eV of tBisICz‐PhCz. In addition, solvatochromism was observed through room temperature PL analysis performed in four solvents with different polarities (Figure S11, Supporting Information). tBisICz‐DPA showed a relatively large red‐shift of peak wavelength and spectral broadening, confirming that it has a slightly stronger CT character than tBisICz‐PhCz. Accordingly, the tBisICz‐PhCz was effective to sharpen the emission spectrum, to reduce the Stokes shift, to intensify absorption, and to purely emit in deep blue region.

**Table 1:** Photophysical properties and electrochemical properties of tBisICz‐DPA and tBisICz‐PhCz

	*λ*_abs_ [nm]	*E*_opt_ ^a)^ [eV]	*λ*_PL_ ^b)^ [nm]	*E*_S_/*E*_T_ ^b)^ [eV]	Δ*E*_ST_ [eV]	Stokes shift^c)^ [nm]	FWHM [nm]
tBisICz‐DPA	279, 361, 427	2.76	444	2.79/2.51	0.28	17	27
tBisICz‐PhCz	273, 360, 428	2.78	440	2.82/2.51	0.31	12	16

^a)^ Calculated from onset energy of UV–vis absorption spectrum;

^b)^ Values at peak position;

^c)^ The difference between the lowest energy absorption peak and peak wavelength of singlet emission.

**Figure 3:**
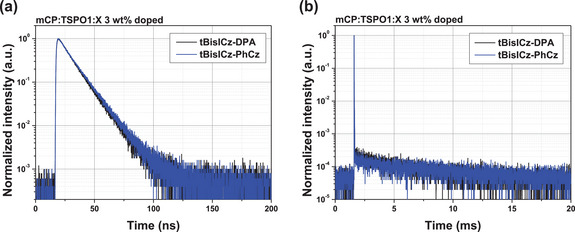
Transient PL decay curve of tBisICz‐DPA (black) and tBisICz‐PhCz (blue) for a) prompt fluorescence component and b) delayed fluorescence component.

The radiative transitions of the emitters were further investigated by measuring PLQY and exciton decay time using the mixed host doped with 3 wt% emitter (**Table** **2**). For efficient energy transfer from host to emitters, 1,3‐di(9*H*‐carbazol‐9‐yl)benzene (mCP) and diphenyl[4‐(triphenylsilyl)phenyl]phosphine oxide (TSPO1) were deposited at a ratio of 50:50 based on weight. The PLQYs of tBisICz‐DPA and tBisICz‐PhCz under nitrogen were 96% and 91%, respectively. Relatively high PLQY was noted in the tBisICz‐DPA by the DPA unit playing a role of auxochromophore. Transient PL analysis provided the prompt decay from intrinsic fluorescence of the emitters and delayed decay from SVC‐TADF mechanism (**Figure** **3**). The temperature‐dependent transient PL analysis was conducted to characterize TADF characteristic of tBisICz‐DPA and tBisICz‐PhCz (Figure S12, Supporting Information). Both compounds clearly exhibited typical TADF behavior showing intensified delayed fluorescence component by increasing temperature. The prompt/delayed decay times of tBisICz‐DPA and tBisICz‐PhCz were 10.7 ns/3.39 ms and 11.5 ns/6.27 ms, respectively. The prompt decay time was of little discrepancy, but the delayed decay time was shortened in the tBisICz‐DPA emitter due to the small Δ*E*_ST_ for efficient up‐conversion. In addition, the delayed decay time of tBisICz‐DPA was obviously shorter than that of tBisICz, indicating that the SVC‐RISC process is accelerated by the donor substitution.

**Table 2:** Photophysical parameters of tBisICz‐DPA and tBisICz‐PhCz doped mCP:TSPO1 film at 3 wt% doping concentration

	*λ*_PL_	FWHM	*Φ*_p_ ^a)^	*Φ*_d_ ^b)^	*τ*_p_ ^c)^	*k*_p_ ^e)^	*τ*_d_ ^d)^	*k*_d_ ^f)^	*k*_ISC_ ^g)^	*k*_RISC_ ^h)^	*k* ^S^_r_ ^i)^	*k* ^T^_nr_ ^j)^
	[nm]	[nm]	[%]	[%]	[ns]	[10^7^ s^−1^]	[ms]	[10 s^−1^]	[10^7^ s^−1^]	[10^3^ s^−1^]	[10^7^ s^−1^]	[10 s^−1^]
tBisICz‐DPA	447	28	45	51	10.7	9.35	3.39	29.5	5.14	0.61	4.21	2.15
tBisICz‐PhCz	443	19	45	46	11.5	8.70	6.27	15.9	4.78	0.30	3.91	2.61

^a)^ PLQY of prompt fluorescence;

^b)^ PLQY of delayed fluorescence;

^c)^ Decay time of prompt fluorescence;

^d)^ decay time of delayed fluorescence;

^e)^ Rate constant of prompt fluorescence;

^f)^ Rate constant of delayed fluorescence;

^g)^ Rate constant of intersystem crossing;

^h)^ Rate constant of reverse intersystem crossing;

^i)^ Rate constant of radiative decay for singlet state;

^j)^ Rate constant of non‐radiative decay for triplet state.

**Figure 4:**
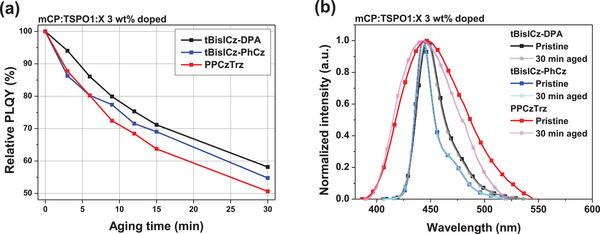
a) Relative PLQY change of the emitter doped films according to UV irradiation time and b) Normalized solid PL spectra before and after UV irradiation.

Furthermore, photostability test of the emitters was performed to discuss the exciton stability of deep blue emitters. In addition to tBisICz‐DPA and tBisICz‐PhCz, 9‐(5′‐(4,6‐diphenyl‐1,3,5‐triazin‐2‐yl)‐[1,1′:3′,1′'‐terphenyl]‐2′‐yl)‐3,6‐diphenyl‐9*H*‐carbazole (PPCzTrz), a donor‐acceptor type TADF material, was also analyzed because it was reported as an electrically and chemically stable TADF emitter with a peak wavelength of 444 nm.^[^ ^24^ ^]^ The photostability of three emitters was evaluated by measuring PLQY according to UV irradiation time. **Figure** **4**a and Figure S13, Supporting Information, provide the relative PLQY drop and the solid PL spectra according to UV irradiation time of up to 30 min. The relative PLQY after aging for 30 min was 58% for tBisICz‐DPA, 55% for tBisICz‐PhCz, and 51% for PPCzTrz, respectively. Figure 4b provides the solid PL spectra before and after UV aging. The *λ*_PL_/FWHM values obtained from pristine films of solid PL spectra were 447 nm/28 nm for tBisICz‐DPA and 443 nm/19 nm for tBisICz‐PhCz, respectively, which were well maintained even after aging. However, a dramatic change of the emission spectrum was noted in the PPCzTrz film after aging. This result demonstrates that the tBisICz derivatives provide better exciton stability than PPCzTrz under UV aging.

**Figure 5:**
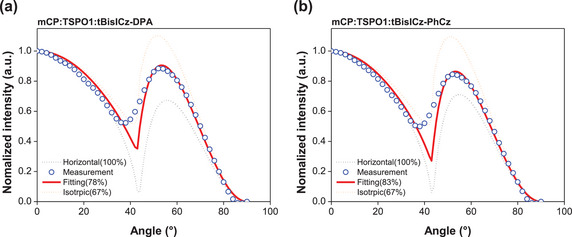
Angle‐dependent PL spectra of a) tBisICz‐DPA and b) tBisICz‐PhCz.

Angle‐dependent PL analysis was also conducted to study the emitting dipole orientation of the emitters and the analysis results are shown in **Figure** **5**. The horizontal emitting dipole orientation ratios of tBisICz‐DPA and tBisICz‐PhCz in the mCP:TSPO1 host were 78% and 83%, respectively. The linear and planar feature of the tBisICz core delivered the high emitting dipole orientation ratio.

### 2.3 Electrochemical and Thermal Analysis

The electrochemical properties were characterized using cyclic voltammetry. The HOMO and LUMO levels were determined by electrochemical oxidation potential and HOMO–LUMO gap obtained from UV–vis absorption. The oxidation scans in Figure S14, Supporting Information, offered the HOMO levels of – 5.65 and – 5.67 eV in the tBisICz‐DPA and tBisICz‐PhCz from the onset potential. As the HOMO is governed by the tBisICz core, similar HOMO level was noted in the two emitters. The electron‐rich DPA group slightly destabilized the HOMO level of tBisICz‐DPA. The LUMO levels of both molecules calculated by HOMO–LUMO gap from UV–vis absorption and LUMO levels were – 2.89 eV. To characterize thermal property, thermogravimetric analysis was conducted to measure the decomposition temperature (*T*_d_) at a 5% weight loss (Figure S15, Supporting Information). The *T*_d_s were determined to be 466 °C for tBisICz‐DPA and 534 °C for tBisICz‐PhCz, which were higher than that of tBisICz. The bulky aromatic group made the main chromophore more thermally robust, and the trend of *T*_d_ followed the order of molecular weight. Therefore, both MR‐TADF compounds could be safely deposited in a vacuum deposition process with little decomposition.

### 2.4 Device Analysis

**Figure 6:**
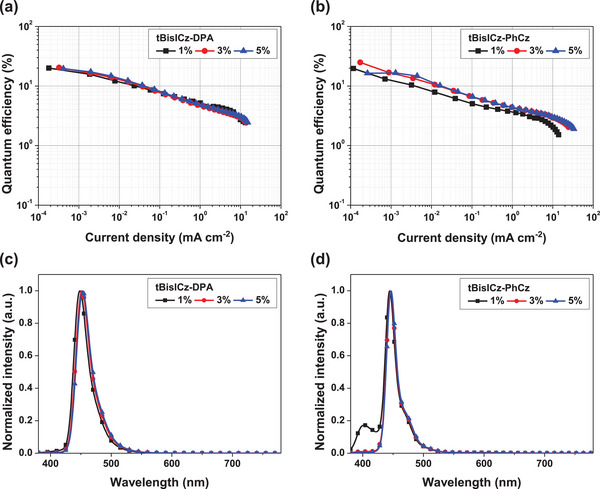
a,b) External quantum efficiency–current density curves and c,d) normalized electroluminescence spectra of tBisICz‐DPA and tBisICz‐PhCz devices at different doping concentration.

The MR‐TADF emitters were embedded in a device structure confining high energy triplet excitons that are necessary for deep blue emitters to evaluate the device performances. Device performance data are summarized in **Table** **3**. The triplet exciton harvesting host materials comprises a mixture of mCP and TSPO1, which are also used as the charge transport materials. The materials and device architecture including the energy level diagram are presented in Figure S16, Supporting Information. The emitters were doped in the mCP:TSPO1 host at doping concentrations of 1, 3 and 5 wt%. Voltage sweep measurement of the device data was conducted to analyze the current density and luminance dependence on the applied voltage in Figure S17, Supporting Information. The current density of the device was little affected by the doping concentration in the tBisICz‐PhCz device, while it was governed by doping concentration in the tBisICz‐DPA. This can be explained by hole trapping function of the tBisICz‐DPA emitter. The hole trapping retarded the hole transport and reduced the current density in the tBisICz‐DPA device. The EQE of the two emitters was plotted against current density for various doping concentration in **Figure** **6**a,b. The EQE of tBisICz‐PhCz devices at 1 wt% doping concentration was relatively low due to incomplete energy transfer as shown in the electroluminescence (EL) spectra (Figure 5d). Whereas, the energy transfer became complete at 3 wt% doping concentration, improving the EQE. The maximum EQEs achieved at 3 wt% doping concentration were 20.4% and 24.9% in the tBisICz‐DPA and tBisICz‐PhCz devices, respectively. Although the PLQY of tBisICz‐DPA was higher than that of tBisICz‐PhCz, tBisICz‐PhCz device recorded higher EQE because tBisICz‐PhCz device had a higher light extraction efficiency originated from high emitting dipole orientation.^[^ ^25^, ^26^ ^]^ Other device performances are in Figure S18, Supporting Information, and Table 3 in comparison with those of tBisICz.

**Table 3:** Summarized device performance data of deep blue MR‐TADF OLEDs

Emitter	Doping conc.	*λ*_EL_ ^a)^ [nm]	FWHM^a)^ [nm]	CIE [*x*, *y*]^a)^	EQE_Max_ [%]	PE_Max_ [lm W^−1^]	CE_Max_ [Cd A^−1^]
tBisICz	3 wt%	448	35	(0.15, 0.07)	18.6	15.2	15.5
tBisICz‐DPA	1 wt%	448	28	(0.15, 0.05)	20.0	10.8	11.0
	3 wt%	452	28	(0.15, 0.05)	20.4	12.1	12.3
	5 wt%	453	29	(0.15, 0.06)	19.5	11.9	12.1
tBisICz‐PhCz	1 wt%	445	20	(0.17, 0.05)	19.7	8.4	8.5
	3 wt%	446	19	(0.16, 0.04)	24.9	11.4	11.6
	5 wt%	447	19	(0.15, 0.04)	16.6	7.5	7.7

^a)^ Values at 100 cd m^−2^.

A unique feature observed in the device data of tBisICz‐PhCz is the constant EL spectra regardless of the doping concentration (Figure 6d). The bulky PhCz unit effectively suppressed the intermolecular packing, minimizing the spectral shift and EQE degradation at high doping concentration. A narrow emission spectrum with FWHM of 19 nm was demonstrated at all doping concentrations, affording an deep blue Commission Internationale d'Eclairage (CIE) color coordinate of (0.16, 0.04) in the tBisICz‐PhCz device. For tBisICz devices, the sharp EL spectrum at low doping concentration was seriously red‐shifted and widened by planar molecular structure (Figure S19, Supporting Information). The DPA blocking group was not as effective as the PhCz unit, but it also exhibited suppressed spectral shifts at high doping concentrations (Figure 6c). Approximately 5 nm shift of the peak wavelength was observed at high doping concentration in the tBisICz‐DPA device due to weak CT nature and relatively incomplete core‐shielding. The FWHM and CIE color coordinate of the tBisICz‐DPA device were 28 nm and (0.15, 0.05) at 3% doping concentration. To demonstrate the doping concentration resistant EL emission characteristics, the devices with 15 wt% doping concentration were fabricated and the device data are summarized in Figure S20 and Table S3, Supporting Information. Despite the high doping concentration, excimer formation was completely suppressed in both cases, and a unique emission spectrum of each MR‐TADF emitter was observed. In particular, the tBisICz‐PhCz device exhibited remarkable emission characteristics in terms of *λ*_EL_, FWHM, and CIE coordinates compared to the 3 wt% emitter doped device by efficient intermolecular interaction control. On the other hand, the tBisICz‐DPA device showed spectral broadening and red‐shifted emission, which well matched the trends of the 1, 3 and 5 wt% emitter doped devices. The device stability of MR‐TADF OLEDs measured under constant current density at an initial luminance of 100 cd m^−2^ is illustrated in Figure S21, Supporting Information. The short device lifetime was observed due to poor material stability of host and charge transport layer under high electrical stress. As a result, a pure deep blue OLED with high efficiency and good color stability up to 15 wt% doping concentration could be realized by introducing a blocking donor at the *ortho*‐position of N atoms (Figure S22, Supporting Information). Maximum EQEs of deep blue OLEDs of this work and previous works are described in Figure S23, Supporting Information. Among them, tBisICz‐PhCz device recorded one of the highest EQE of deep blue OLEDs satisfying the BT.2020 color standard. The rigid MR structure and carbazole blocking group enabled the high EQE of 24.9%, small FWHM of 19 nm, CIE*_y_* of 0.04 and doping concentration resistant device performances.

## 3 Conclusions

In this work, highly efficient and deep blue MR TADF emitters with a tBisICz core and DPA or phenylcarbazole blocking group were designed and evaluated for deep blue TADF OLEDs. The blocking group effectively suppressed intermolecular interaction without disturbing the emission properties of the MR‐TADF emitters. In particular, phenylcarbazole modified tBisICz‐PhCz showed small Stokes shift of 12 nm, high PLQY of 91% and small FWHM of 16 nm while showing a horizontal emitting dipole orientation ratio of 83%. As a result, the MR‐TADF OLED device with the tBisICz‐PhCz recorded deep blue emission with small FWHM of 19 nm, EQE of 24.9%, and CIE coordinate of (0.16, 0.04) without any spectral shift according to doping concentration. We believe that the MR‐TADF design introducing tBisICz core and phenylcarbazole blocking group can be effectively applied to highly efficient deep blue emitters satisfying BT.2020 standard.

## Conflict of Interest

The authors declare no conflict of interest.

## Supporting information

Supporting Information

## Data Availability

Research data are not shared.
