# Supplementary material for: Color Stable Deep Blue Multi‐Resonance Organic Emitters with Narrow Emission and High Efficiency
Source: Adv Sci (Weinh). 2023 Jul 9;10(26):2302619. doi: 10.1002/advs.202302619 (PMC10502835; doi:10.1002/advs.202302619)
Supplement: Supplementary file 1 — Supporting Information [file ADVS-10-2302619-s001.pdf]

ADVANCED SCIENCE
Open Access

Supporting Information

for *Adv. Sci.*, DOI 10.1002/advs.202302619

Color Stable Deep Blue Multi-Resonance Organic Emitters with Narrow Emission and High Efficiency

Jihoon Kang, Soon Ok Jeon, Inkoo Kim, Ha Lim Lee, Junseop Lim and Jun Yeob Lee**

Supporting Information

Color Stable Deep Blue Multi-Resonance Organic Emitters with Narrow Emission and High Efficiency

Jihoon Kang[1], Soon Ok Jeon[2*], Inkoo Kim[3], Ha Lim Lee[1], Junseop Lim[1], Jun Yeob Lee[1,4,5*]

[1]School of Chemical Engineering, Sungkyunkwan University

2066, Seobu-ro, Jangan-gu, Suwon, Gyeonggi, 16419, Republic of Korea

[2]Samsung Advanced Institute of Technology, Samsung Electronics Co., Ltd.,

130 Samsung-ro, Yeongtong-gu, Suwon, Gyeonggi, 16678, Republic of Korea

[3]Innovation Center, Samsung Electronics Co., Ltd.,

Hwaseong, Gyeonggi, 18448, Republic of Korea

[4]SKKU Advanced Institute of Nano Technology, Sungkyunkwan University

2066, Seobu-ro, Jangan-gu, Suwon, Gyeonggi, 16419, Republic of Korea

[5]SKKU Institute of Energy Science and Technology, Sungkyunkwan University

2066, Seobu-ro, Jangan-gu, Suwon, Gyeonggi, 16419, Republic of Korea

E-mail : leej17@skku.edu, so.jeon@samsung.com

1. General information

2. Methods

2.1. Synthesis

2.2. Electronic structure calculations

2.3. Photophysical characterization

2.3.1. Ultraviolet-visible spectroscopy and photoluminescence analysis

2.3.2. Transient photoluminescence and absolute photoluminescence quantum yield

2.4. Electrochemical and thermal analysis

2.5. Device analysis

3. Supplementary table

Supplementary Reference

1. General information

3,6-Di-*tert*-butyl-9*H*-carbazole, 1,4-dibromo-2,5-diiodobenzene and bis(pinacolato) diboron were purchased from INCO Co. (9-Phenyl-9*H*-carbazol-3-yl)boronic acid was obtained from GOM Tech Co. 2-Dicyclohexylphosphino-2′,6′-dimethoxybiphenyl (SPhos), tetrakis(triphenylphosphine)palladium(0) ($Pd(PPh_3)_4$), tris(dibenzylideneacetone)dipalladium and [1,1′-bis(diphenylphosphino)ferrocene]dichloropalladium(II) ($Pd_2(dba)_3$) were purchased from P&H Tech Co. 2-Dicyclohexylphosphino-2′,4′,6′-triisopropylbiphenyl (XPhos) was obtained from SamYoung Innovation Co.. Diphenylamine, sodium *tert*-butoxide, copper(I) iodide (CuI), *N*-bromosuccinimide, and 1,10-phenanthroline were purchased from Alfa Aesar and Thermo Fisher Scientific Inc. Sodium thiosulfate, potassium acetate (KOAc), potassium phosphate tribasic (K_3PO_4), potassium carbonate (K_2CO_3), magnesium sulfate anhydrous ($MgSO_4$), celite, *N*,*N*-dimethylformamide (DMF), 1,2-dimethoxyethane (1,2-DME) and 1,4-dioxane were purchased from Daejung Chemical & Metal Co. Methylene chloride (MC), toluene and tetrahydrofuran (THF) were obtained from Samchun Pure Chemical Co.

The structural information of synthesized compounds was obtained by nuclear magnetic resonance (NMR) and mass spectroscopy. The NMR spectra were obtained from Oxford 300 NMR (VARIAN, 300 MHz), Unity Inova (VARIAN, 500 MHz) and ASCEND 500 (Bruker, 500 MHz) using DMSO-d_6, $CDCl_3$ and CD_2Cl_2. For mass spectroscopy, Advion, Expresion^L CMS spectrometer using atmospheric pressure chemical ionization (APCI) mode and the liquid chromatography mass spectrometer-ion trap-time of flight (LCMS-IT-TOF) Shimadzu spectrometer were used. The LCMS equipment was operated using mobile phase of a mixed solvent of acetonitrille and water at a flow rate of 0.5 mL/min. Sampling solvent was anhydrous THF. APCI ionization source was used.

2. Methods

2.1. Synthesis

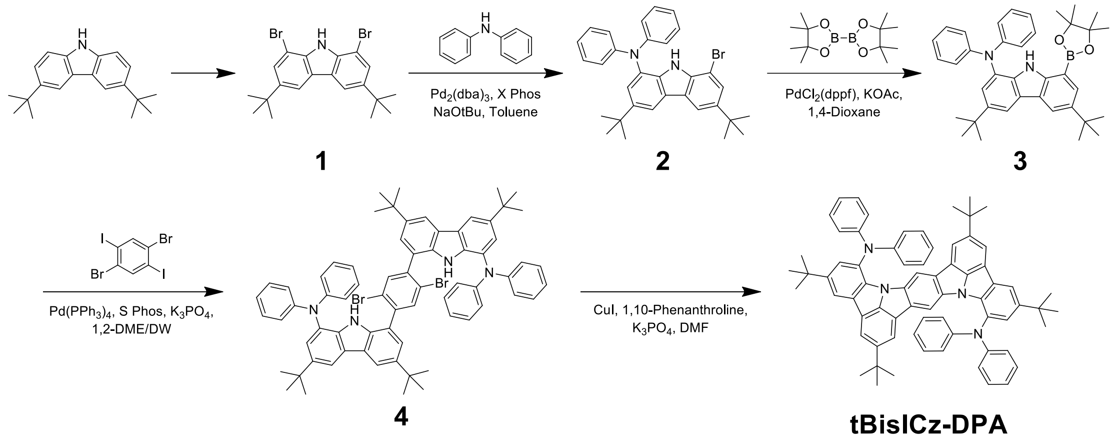

Scheme S1. Synthetic scheme of tBisICz-DPA.

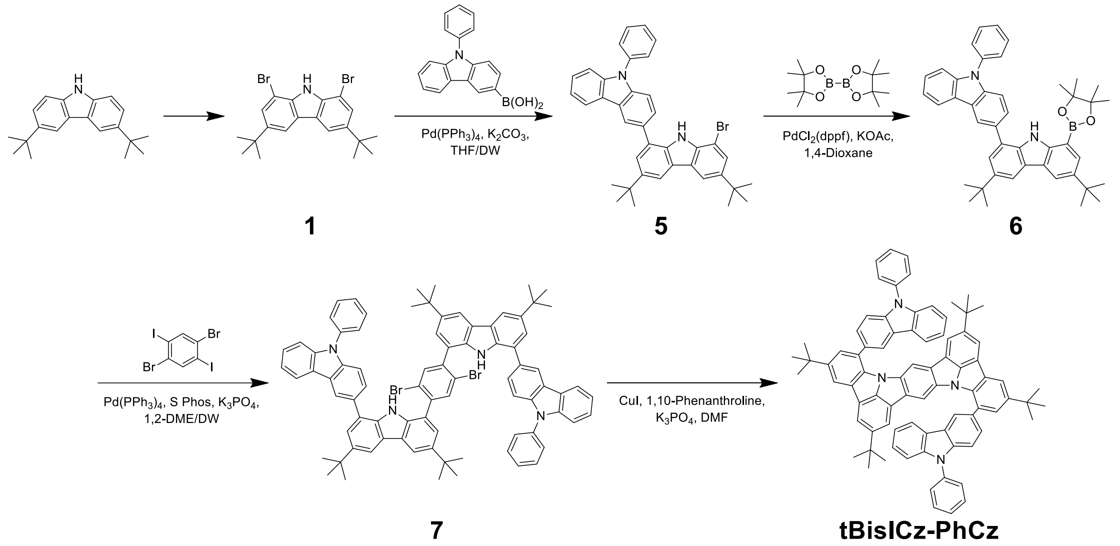

Scheme S2. Synthetic scheme of tBisICz-PhCz.

We were synthesized 1,8-dibromo-3,6-di-*tert*-butyl-9*H*-carbazole (**1**) according to the synthetic method of the previously reported paper.[S1]

8-Bromo-3,6-di-*tert*-butyl-*N*,*N*-diphenyl-9*H*-carbazol-1-amine (2)

Diphenylamine (1.50 g, 8.86 mmol), 1,8-dibromo-3,6-di-*tert*-butyl-9*H*-carbazole (**1**) (9.69 g, 22.16 mmol), tris(dibenzylideneacetone)dipalladium ($Pd_2(dba)_3$, 0.24 g, 0.27 mmol), XPhos (0.25 g, 0.53 mmol) and sodium *tert*-butoxide (NaOtBu,1.70 g, 17.73 mmol) were added into a 250 mL 2-neck round-bottomed (RB) flask followed by slow addition of toluene (80 mL). The reaction mixture was stirred and refluxed during 18 h under a nitrogen atmosphere. After completion of the reaction, the reaction mixture was slowly cooled to room temperature and diluted with 160 mL of MC then, the mixture was filtered with celite/silica gel filter and concentrated through a rotary evaporator. The mixture was purified through column chromatography with an eluent of MC : *n*-hexane (1:1). The product was obtained as a white solid. (2.42 g, Yield 52%)

^{1}H NMR (300 MHz, DMSO-d_6) : δ 10.08 (s, 1H), 8.22(s, 1H), 8.15 (s, 1H), 7.54 (s, 1H), 7.30 (s, 1H), 7.24 (t, J = 7.2 Hz, 4H), 7.04(d, J = 8.2Hz, 4H), 6.94 (t, J = 7.2 Hz, 2H), 1.32 (s, 9H), 1.32 (s, 9H)

MS (APCI) m/z : Found 525.09 [$(M + H)^+$]. Calculated For $C_{32}H_{33}BrN_2$: 524.18.

3,6-Di-*tert*-butyl-*N*,*N*-diphenyl-8-(4,4,5,5-tetramethyl-1,3,2-dioxaborolan-2-yl)-9*H*-carbazol-1-amine (3)

8-Bromo-3,6-di-*tert*-butyl-*N*,*N*-diphenyl-9*H*-carbazol-1-amine (**2**) (1.50 g, 2.85 mmol), bis(pinacolato) diboron (1.45 g, 5.71 mmol), $Pd_2(dba)_3$ (0.06 g, 0.09 mmol), and KOAc (0.56 g, 5.71 mmol) were added into a 100 mL 2-neck RB flask followed by addition of 1,4-dioxane (20 mL). The reaction mixture was refluxed for 14 h under a nitrogen atmosphere. After completion of the reaction, the reaction mixture was slowly cooled to room temperature and diluted with 70 mL of MC then, the mixture

was filtered with celite/silica gel filter and concentrated through a rotary evaporator. The mixture was purified through column chromatography with an eluent of MC : *n*-hexane (1:1). The product was obtained as a white powder. (1.11 g, Yield 68%)

^{1}H NMR (300 MHz, DMSO-d_6) : δ 8.41 (s, 1H), 8.36 (s, 1H), 8.07 (s, 1H), 8.07 (s, 1H), 7.62 (s, 1H), 7.30 (t, *J* = 7.2 Hz, 4H), 7.17 (s, 1H), 7.05-6.99 (m, 6H), 1.37 (s, 9H), 1.31 (s, 9H), 1.20 (s, 12H)

MS (APCI) m/z : Found 573.25 [(M + H)$^+$]. Calculated For $C_{38}H_{45}BN_2O_2$: 572.36

8,8'-(2,5-Dibromo-1,4-phenylene)bis(3,6-di-*tert*-butyl-*N*,*N*-diphenyl-9*H*-carbazol-1-amine) (4)

1,4-Dibromo-2,5-diiodobenzene (0.27 g, 0.56 mmol), Pd(PPh_3)$_4$ (0.07 g, 0.06 mmol), and SPhos (0.05 g, 0.11 mmol) were added into a 50 mL 2-neck RB flask and 2 mL of 1,2-DME was slowly dropped to the RB. And then, the 4M solution of K_3PO_4 (2.55 g, 12.00 mmol) was dissolved into deionized water (DW) was added into the mixture. The reactants were stirred and slightly heated up to 60 °C under a nitrogen condition for 10 min. The solution of 3,6-Di-*tert*-butyl-*N*,*N*-diphenyl-8-(4,4,5,5-tetramethyl-1,3,2-dioxaborolan-2-yl)-9*H*-carbazol-1-amine (**3**) (0.80 g, 1.38 mmol) dissolved into 1,2-DME (16 mL) was carefully dropped into the reaction mixture. The reaction was was kept overnight at 80 °C, allowing the mixture to slowly warm up to room temperature. The mixture was extracted with MC and concentrated through a rotary evaporator. The mixture was adsorbed on silica gel and purified through column chromatography with an eluent of MC : *n*-hexane (1:4). The separated product was dissolved in MC and re-precipitated using methanol. The precipitated product was obtained as a white powder by filtering and drying in vacuum oven. (0.14 g, Yield 22%)

^{1}H NMR (300 MHz, $CDCl_3$) δ 8.14 (s, 2H), 7.96 (s, 2H), 7.49 (s, 2H), 7.39 (s, 2H), 7.32 (s, 2H), 7.24 – 7.16 (m, 5H), 7.15 – 7.10 (m, 5H), 7.10 – 7.00 (m, 8H), 6.98 – 6.88 (m, 4H), 1.50 (s, 18H), 1.40 (s, 18H).

LC/MS (APCI) m/z : Found 1123.00 [$(M)^+$]. Calculated For $C_{70}H_{68}Br_2N_4$: 1122.38

2,5,11,14-Tetrakis(1,1-dimethylethyl)-*N*^{7},*N*^{7},*N*^{16},*N*^{16}-tetraphenyl-indolo[3,2,1-*jk*]-indolo[1',2',3':1,7]indolo[3,2-*b*]carbazol-7,16-amine (tBisICz-DPA)

8,8'-(2,5-Dibromo-1,4-phenylene)bis(3,6-di-*tert*-butyl-*N*,*N*-diphenyl-9*H*-carbazol-1-amine) (**4**) (0.13 g, 0.12 mmol), CuI (0.02 g, 0.12 mmol), 1,10-phenanthroline (0.02 g, 0.12 mmol), and K_3PO_4 (0.10 g, 0.46 mmol) were added into a 100 mL pressure sealed tube followed by addition of DMF (5 mL). The reaction was carried out for 24 h at 100 ℃. After cooling, the mixture was diluted with MC and filtered with celite/silica gel filter. The filtrate was concentrated through a rotary evaporator and adsorbed on silica gel. The product was purified using column chromatography with an eluent of MC : *n*-hexane (1:4). The separated product was dissolved into MC and methanol was added for precipitation. After drying in the vacuum oven, a yellow powder was obtained (0.09 g, Yield 81%). Further purification was done by train sublimation for vacuum deposition.

^{1}H NMR (500 MHz, CD_2Cl_2) : δ 8.57 (s, 2H), 8.19 (d, *J* = 1.1 Hz, 2H), 8.16 (d, *J* = 1.9 Hz, 2H), 7.92 (d, *J* = 1.1 Hz, 2H), 7.41 (d, *J* = 1.9 Hz, 2H), 7.32-7.30 (m, 8H), 7.28-7.25 (m, 8H), 6.96-6.95 (m, 4H), 1.58 (s, 18H), 1.40 (s, 18H)

^{13}C NMR (500 MHz, CD_2Cl_2) : δ 147.31, 146.39, 146.30, 143.72, 136.21, 134.58, 131.74, 130.40, 128.99, 128.89, 126.43, 121.62, 120.98, 118.12, 117.93, 117.51, 115.87, 115.77, 108.08, 35.34, 34.33, 32.09, 31.03

HRMS (FAB) m/z : Found 962.5279 [(M)$^+$]. Calculated For $C_{70}H_{66}N_4$: 962.5287. EA (%) : Found C, 86.7, H, 6.9, N, 5.9. Calculated For : C, 87.28, H, 6.91, N, 5.82.

8-Bromo-3,6-di-*tert*-butyl-9'-phenyl-9*H*,9'*H*-1,3'-bicarbazole (5)

1,8-Dibromo-3,6-di-*tert*-butyl-9*H*-carbazole (**1**) (7.61 g, 17.41 mmol), (9-phenyl-9*H*-carbazol-3-yl)boronic acid (2.00 g, 6.97 mmol), $Pd(PPh_3)_4$ (0.24 g, 0.21 mmol), and THF (60 mL) were added into a 2-neck RB flask followed by addition of 4 M aqueous K_2CO_3 (8.29 g, 60.00 mmol) solution. Under a nitrogen atmosphere, the mixture was refluxed for 8 h. After cooling down, the mixture was extracted with MC and DW. The residual moisture in separated organic phase was removed with anhydrous $MgSO_4$. The concentrated mixture was adsorbed on silica gel, and product was separated through column chromatography using an eluent of MC : Hex (1:6). The product was obtained as a white solid. (2.50 g, Yield 60%)

^{1}H NMR (300 MHz, DMSO-d_6) : δ 10.32 (s, 1H), 8.55 (s, 1H), 8.36 (d, J=7.7 Hz, 1H), 8.26 (d, *J* = 1.6 Hz, 1H), 8.22 (d, *J* = 1.7 Hz, 1H), 7.77-7.65 (m, 5H), 7.61-7.55 (m, 4H), 7.47-7.41 (m, 2H), 7.31 (t, *J* = 7.1 Hz, 1H), 1.46 (s, 9H), 1.40 (s, 9H)

MS (APCI) m/z : Found 599.11 [(M + H)$^+$]. Calculated For $C_{38}H_{35}BrN_2$: 598.20

3,6-Di-*tert*-butyl-9'-phenyl-8-(4,4,5,5-tetramethyl-1,3,2-dioxaborolan-2-yl)-9*H*,9'*H*-1,3'-bicarbazole (6)

3,6-Di-*tert*-butyl-9'-phenyl-8-(4,4,5,5-tetramethyl-1,3,2-dioxaborolan-2-yl)-9*H*,9'*H*-1,3'-bicarbazole (**6**) was prepared following the same synthetic and purification methods used for 3,6-di-*tert*-butyl-*N*,*N*-diphenyl-8-(4,4,5,5-tetramethyl-1,3,2-dioxaborolan-2-yl)-9*H*-carbazol-1-amine (**3**). 8-Bromo-3,6-di-*tert*-butyl-9'-phenyl-9*H*,9'*H*-1,3'-bicarbazole (**5**) (1.20 g, 2.00 mmol), bis(pinacolato) diboron (1.27 g, 5.00

mmol), $Pd_2(dba)_3$ (0.04 g, 0.06 mmol), KOAc (0.39 g, 4.00 mmol), and 1,4-dioxane (14 mL) were used. The product was a white solid. (1.76 g, Yield 68%)

^{1}H NMR (300 MHz, DMSO-d$_6$) : δ 9.59 (s, 1H), 8.70 (s, 1H), 8.44 (s, 1H), 8.39 (d, J = 7.8 Hz, 1H), 8.25 (s, 1H), 7.81 (d, 1H), 7.74-7.68 (m, 5H), 7.63 (t, J = 1.5 Hz, 1H), 7.58 (d, J = 8.5 Hz, 2H), 7.49-7.40 (m, 2H), 7.36-7.29 (m, 1H), 1.49 (s, 9H), 1.42 (s, 9H), 1.27 (s, 12H)

MS (APCI) m/z : Found 647.25 [$(M + H)^+$]. Calculated For $C_{44}H_{47}BN_2O_2$: 646.37

8,8''-(2,5-Dibromo-1,4-phenylene)bis(3,6-di-*tert*-butyl-9'-phenyl-9*H*,9'*H*-1,3'-bicarbazole) (7)

3,6-Di-*tert*-butyl-9'-phenyl-8-(4,4,5,5-tetramethyl-1,3,2-dioxaborolan-2-yl)-9*H*,9'*H*-1,3'-bicarbazole (**6**) (0.80 g, 1.2 mmol), 1,4-dibromo-2,5-diiodobenzene (0.24 g, 0.5 mmol), $Pd(PPh_3)_4$ (0.06 g, 0.05 mmol), SPhos (0.04 g, 0.1 mmol) and 8 mL of 1,2-DME were added into a 2-neck RB flask followed by addition of 4 M aqueous solution of K_3PO_4 (3.40 g, 16.0 mmol). The reaction was carried out for 12 h at 80 °C. The product was purified by the same procedure as that of 8,8'-(2,5-dibromo-1,4-phenylene)bis(3,6-di-*tert*-butyl-*N*,*N*-diphenyl-9*H*-carbazol-1-amine) (4). The purified product was a white solid. (0.23 g, Yield 36%)

^{1}H NMR (500 MHz, CD_2Cl_2) : δ 8.29 – 8.07 (m, 8H), 7.84 (s, 2H), 7.67 – 7.24 (m, 26H), 1.50 (s, 36H).

MS (APCI) m/z : Found 1272.12 [$(M + H)^+$]. Calculated For $C_{82}H_{72}Br_2N_4$: 1270.41

2,5,11,14-Tetrakis(1,1-dimethylethyl)-7,16-bis(9-phenyl-carbazol-3yl)-indolo[3,2,1-*jk*]-indolo[1',2',3':1,7]indolo[3,2-*b*]carbazole (tBisICz-PhCz)

8,8''-(2,5-Dibromo-1,4-phenylene)bis(3,6-di-*tert*-butyl-9'-phenyl-9*H*,9'*H*-1,3'-bicarbazole) (**7**) (0.30 g, 0.24 mmol), CuI (0.05 g, 0.24 mmol), 1,10-phenanthroline (0.04 g, 0.24 mmol), K_3PO_4 (0.20 g, 0.94 mmol) and 4 mL of DMF were added into a 100 mL pressure tube. The reaction was carried out 8 h at 80 °C. The product was purified by the same procedure as that of **tBisICz-DPA**. The final emitter **tBisICz-PhCz** was obtained as a yellow solid powder. (0.22 g, Yield 86%). Further purification was done by train sublimation for vacuum deposition.

^1H NMR (500 MHz, CD_2Cl_2) : δ 8.60 (d, *J* = 1.0 Hz, 5H), 8.56 (d, *J* = 1.2 Hz, 2H), 8.29 (d, *J* = 7.6 Hz, 5H), 8.16 (s, 1H), 8.15 – 8.13 (m, *J* = 1.9 Hz, 8H), 8.04 (d, *J* = 1.0 Hz, 2H), 8.02 (d, *J* = 1.1 Hz, 5H), 7.76 (d, *J* = 7.7 Hz, 4H), 7.73 (d, *J* = 8.3 Hz, 2H), 7.68 (ddd, *J* = 8.3, 3.6, 2.2 Hz, 12.5H, overlapped), 7.65 – 7.60 (m, 20H, overlapped), 7.59 (dd, *J* = 2.7, 1.2 Hz, 6.5H, overlapped), 7.54 (td, *J* = 8.5, 1.6 Hz, 17.5H, overlapped), 7.50 – 7.45 (m, 5H), 7.42 (Tt, *J* = 7.7, 2.3, 1.2 Hz, 5H), 7.36 (Td, 5H), 7.28 – 7.24 (m, 2H), 6.96 (d, *J* = 1.0 Hz, 2H), 6.85 (d, *J* = 1.1 Hz, 5H), 6.48 (s, 2H), 6.18 (s, 5H), 1.49 (s, 45H), 1.48 (s, 18H), 1.16 (s, 45H), 1.03 (s, 18H).

^{13}C NMR (126 MHz, CD_2Cl_2) δ 146.50, 146.34, 144.83, 144.66, 141.48, 141.36, 140.66, 140.58, 137.72, 137.62, 137.13, 136.87, 135.44, 135.38, 133.39, 133.35, 130.35, 130.22, 129.93, 129.13, 128.84, 128.75, 128.61, 128.09, 128.00, 127.51, 127.42, 127.13, 126.98, 126.90, 126.58, 126.45, 123.89, 123.40, 121.80, 121.52, 120.69, 120.54, 120.50, 120.43, 118.63, 118.47, 118.03, 117.99, 117.88, 116.00, 115.08, 114.92, 110.83, 110.66, 109.96, 109.86, 108.09, 107.83, 35.43, 35.30, 34.79, 34.75, 32.42, 32.27, 31.71.

HRMS (FAB) m/z : Found 1110.5593 [$(M)^+$]. Calculated For $C_{82}H_{70}N_4$: 1110.5600.

EA (%) : Found C, 87.8, H, 6.3, N, 5.1. Calculated For : C, 88.61, H, 6.35, N, 5.04.

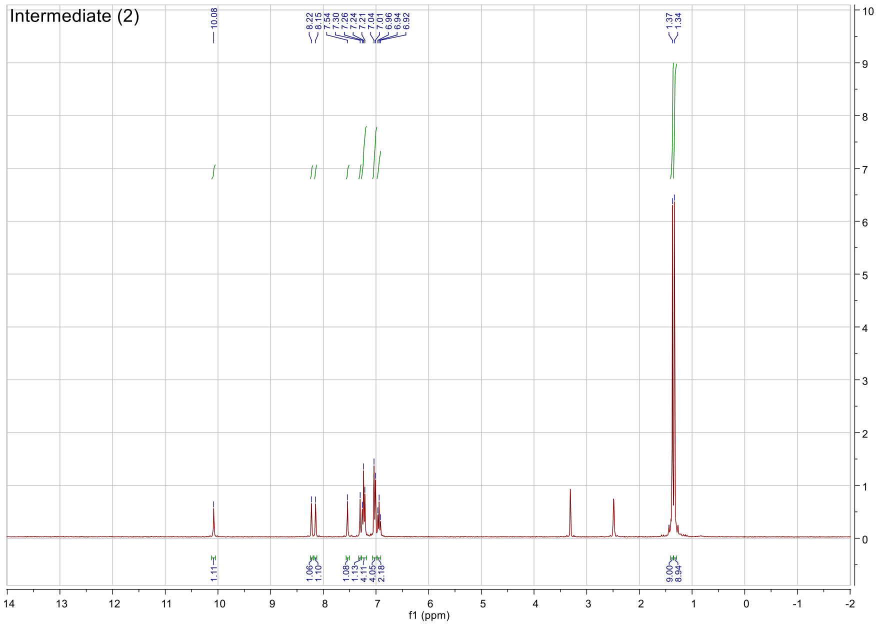

Figure S1. ^{1}**H NMR spectrum of intermediate (2).**

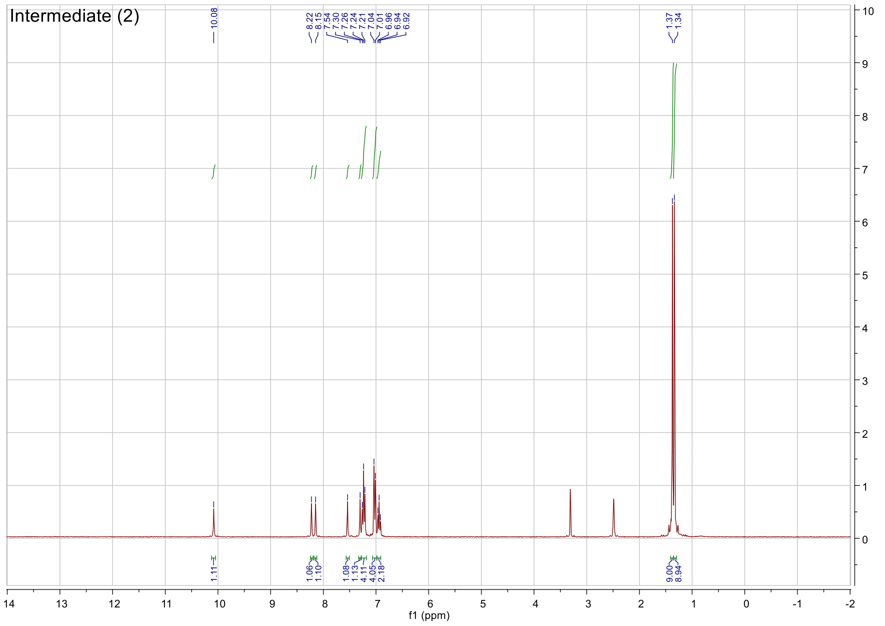

Figure S2. ^{1}**H NMR spectrum of intermediate (3).**

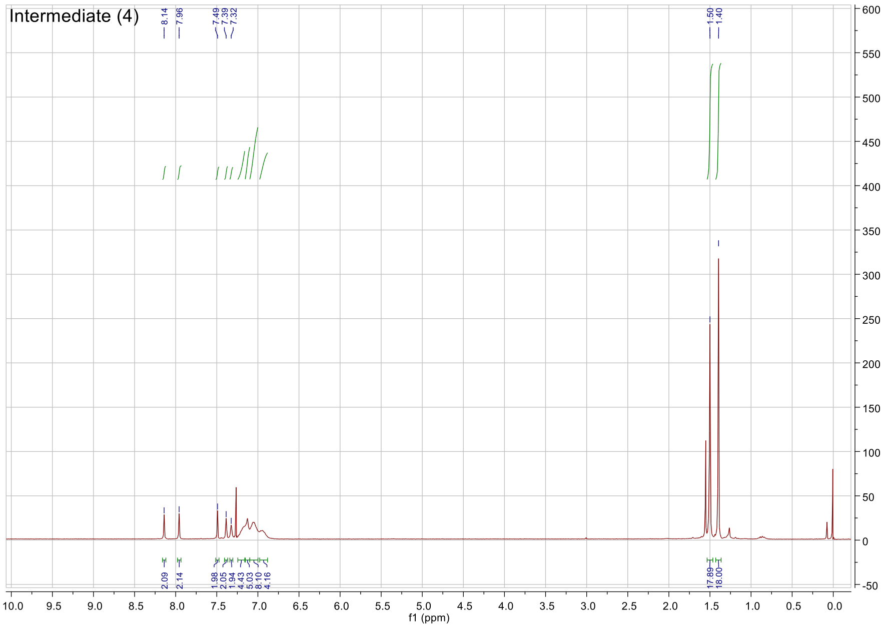

Figure S3. ^{1}H NMR spectrum of intermediate (4).

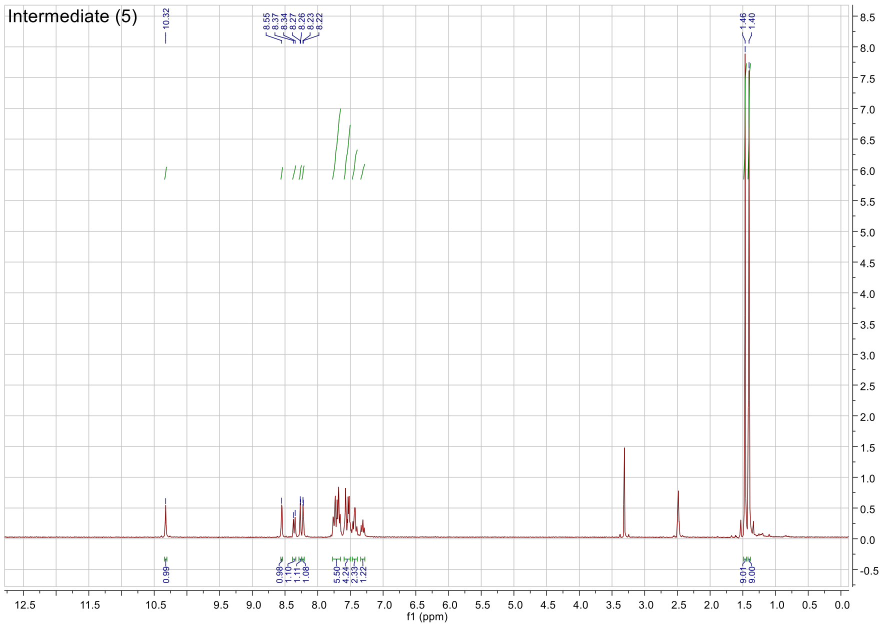

Figure S4. ^{1}H NMR spectrum of intermediate (5).

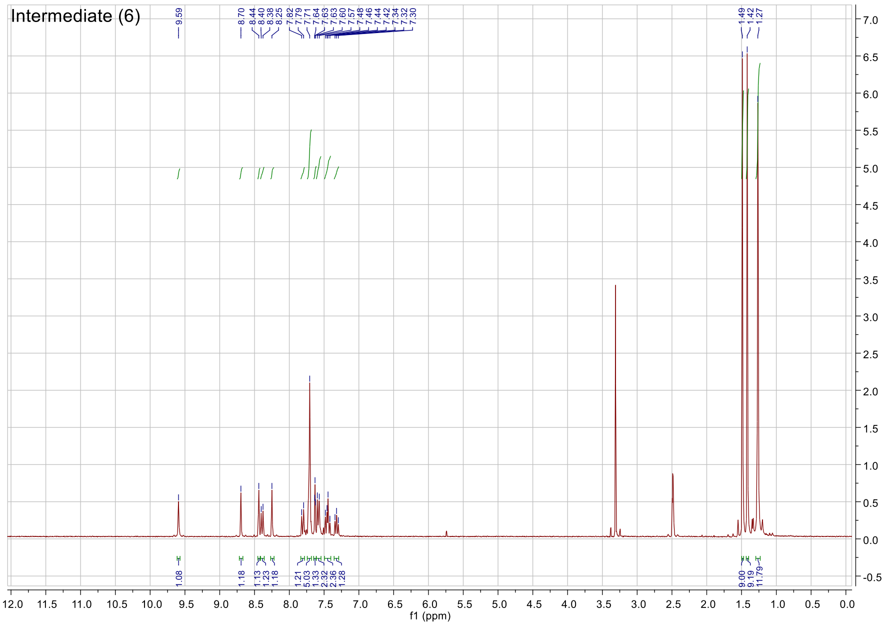

Figure S5. ^{1}H NMR spectrum of intermediate (6).

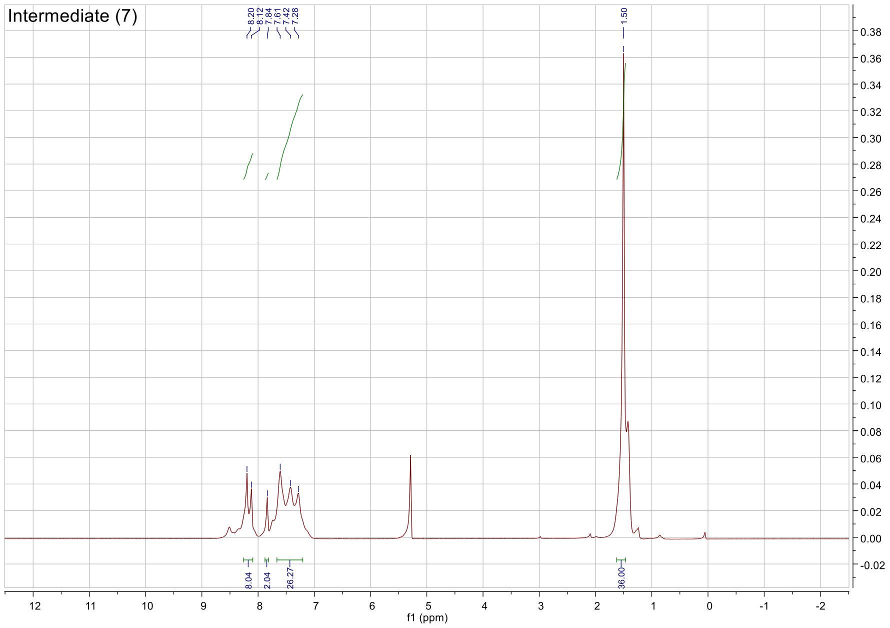

Figure S6. ^{1}H NMR spectrum of intermediate (7).

(a)

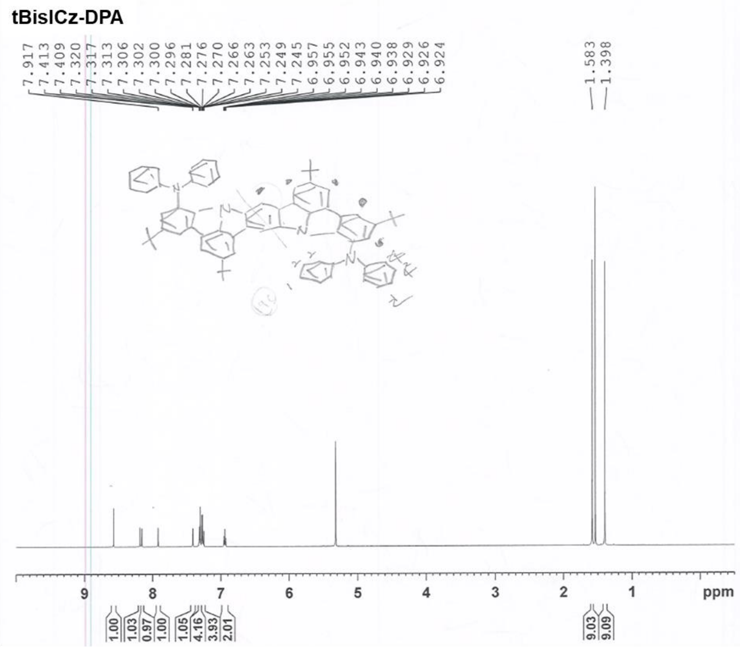

(b)

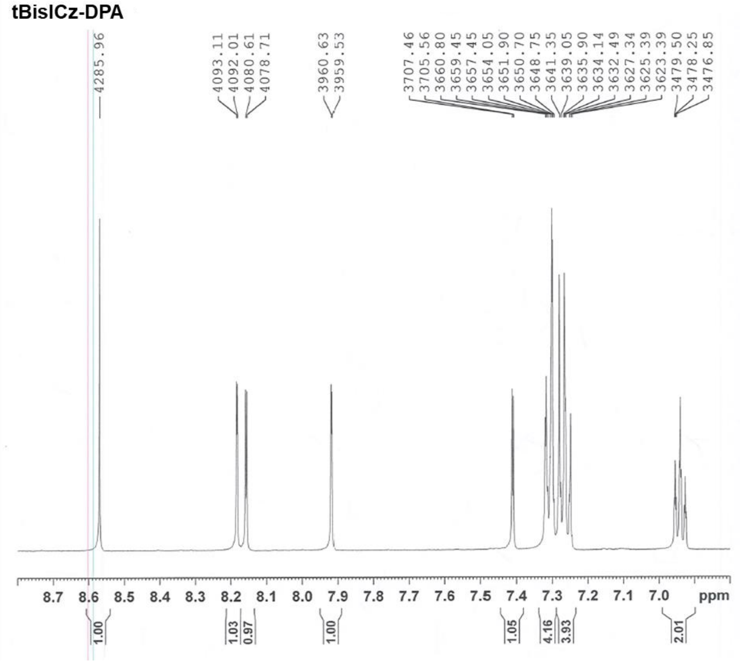

Figure S7. ^{1}H NMR spectrum of tBisICz-DPA. (a) Full range. (b) Enlarged aromatic range.

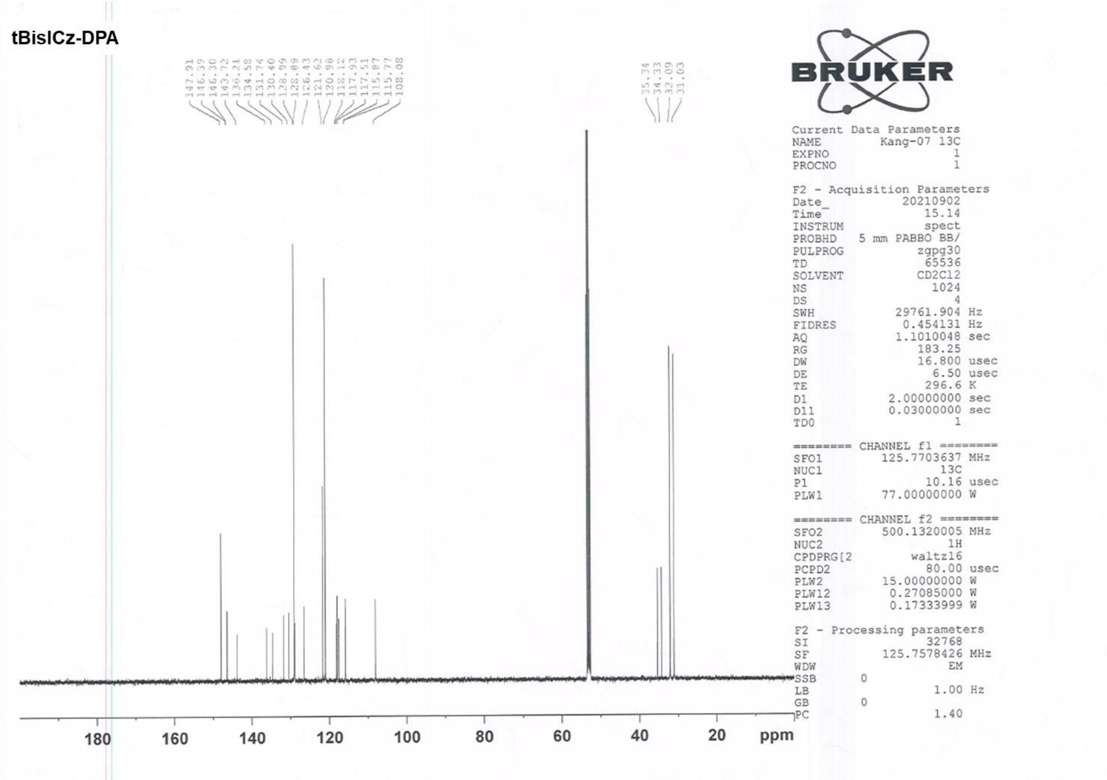

Figure S8. ^{13}**C NMR spectrum of tBisICz-DPA.**

(a)

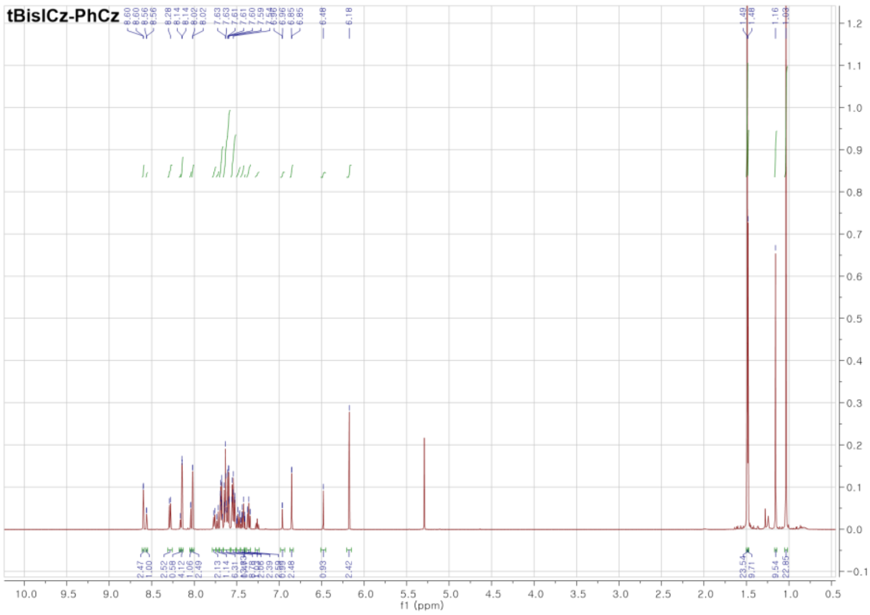

(b)

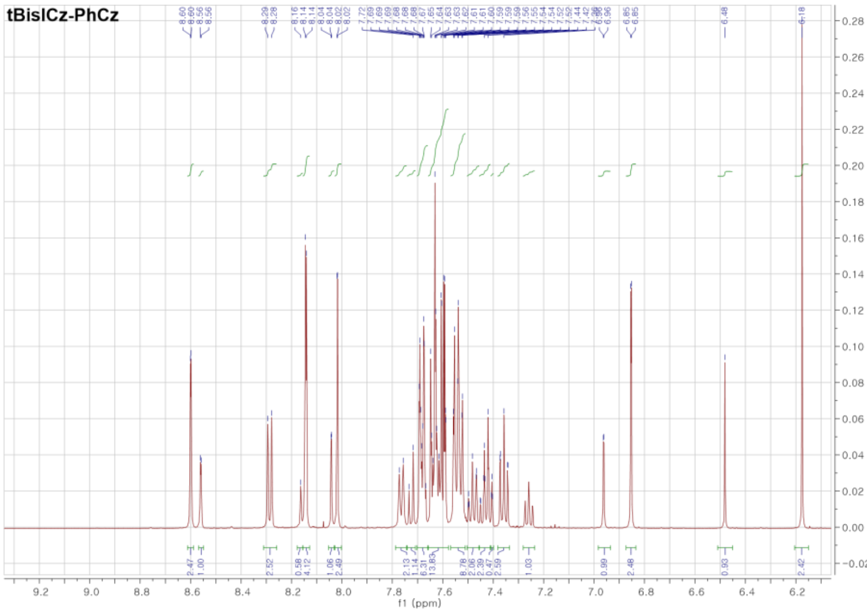

Figure S9. ^{1}H NMR spectrum of tBisICz-PhCz. (a) Full range. (b) Enlarged aromatic range.

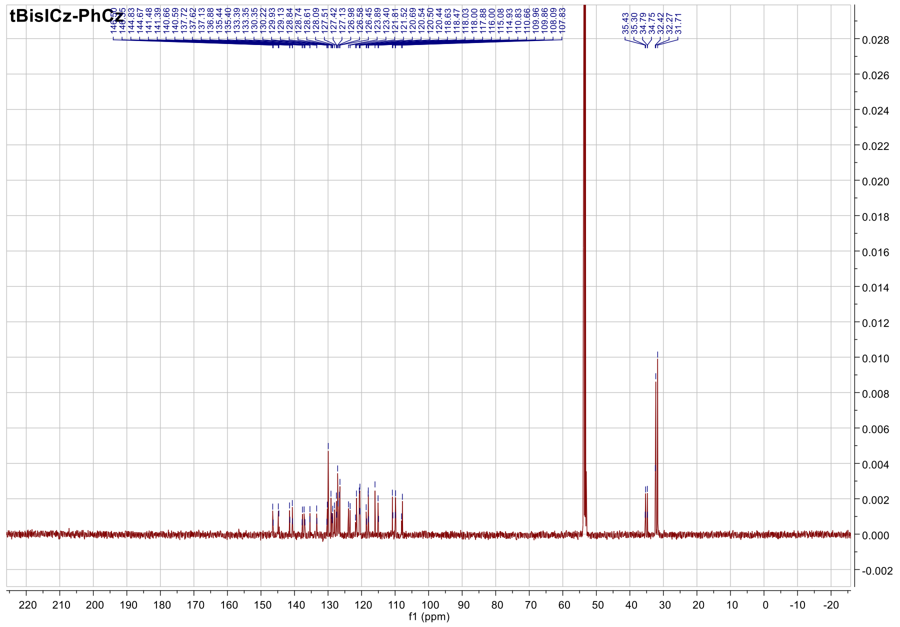

Figure S10. ^{13}C NMR spectrum of tBisICz-PhCz.

2.2. Electronic structure calculations

The excited state geometries of the tBisICz derivatives were obtained using the TURBOMOLE 7.6[S2] program with the time-dependent density functional theory (TDDFT) using the hybrid version of the Becke's three parameter exchange functional with the Lee-Yang-Parr correlation functional (B3LYP)[S3-S6] under the Tamm-Dancoff approximation[S7] by the constrained geometry optimization by fixing the tBisICz core. We employed the tBisICz geometries of the S_1 and T_1 states obtained at the strict variant of the second-order algebraic diagrammatic construction with the spin-component scaling (SCS-ADC(2)) level[S8,S9] and replaced the hydrogens at the 1, 5-positions with DPA or PhCz. Because of the bulkiness of these groups, the geometry optimizations at the cost-expensive ADC(2) level was not feasible, and we had to resort to the TDDFT level; however, as evidenced by the almost unaltered emission spectra after the substitutions discussed in the main text, the excited S_1 geometry is hardly affected by these substitutions. Adiabatic ΔE_{ST} was calculated at these optimized geometries at the SCS-ADC(2) level. Owing to unavailability of spin-orbit coupling and nonadiabatic coupling at the ADC(2) level in the employed programs, these were calculated using the gap-tuned ω^*B97M-V[S10] using the Q-Chem 5.4 program[S11]. The ω^*B97M-V level was also used to calculate the 20 singlet and 20 triplet states that were used in the perturbative expansion in the spin-vibronic Hamiltonian. The one-electron Breit-Pauli Hamiltonian was employed for the spin-orbit coupling. Solvent effects were treated with the polarizable continuum model in the integral equation formalism (IEF-PCM) with the dielectric constant $\epsilon = 2.84$ derived from the archetypal blue host material 1,3-bis(*N*-carbazolyl)benzene (mCP). The reverse intersystem crossing rates were calculated using our in-house code, which is based on the time-correlation function approach for the Fermi golden rule rate equation.[20,21] The time integration interval of [-6553.6:6553.6] fs was used for handling the time-correlation function, which was

discretized with a time step of 12.5 ps. The def2-SVP basis set was used for all electronic structure calculations.

Table S1. Constrained optimized geometries of the S_1 and the T_1 states obtained at the B3LYP/def2-SVP level of theory in Cartesian coordinates (Å) with the tBisICz core optimized at the SCS-ADC(2)/def2-SVP level of theory.

	S_1 statez			T_1 state		
**atom**	**X**	**Y**	**Z**	**X**	**Y**	**Z**
			tBisICz-DPA			
C	1.372121	-0.523292	-0.071343	1.389207	-0.533188	-0.075633
C	0.765107	0.017715	-1.222888	0.756461	0.024883	-1.219502
C	-0.607025	0.556334	-1.103239	-0.655001	0.571344	-1.090122
C	-1.371671	0.523684	0.074082	-1.388993	0.533037	0.078216
C	-0.764686	-0.017399	1.225606	-0.756249	-0.025040	1.222083
C	0.607423	-0.556071	1.105942	0.655215	-0.571495	1.092704
N	-0.954467	1.037557	-2.353999	-0.989313	1.052366	-2.343450
C	0.116742	0.831485	-3.201927	0.085246	0.848451	-3.184846
C	1.212633	0.228742	-2.585740	1.186154	0.238439	-2.573172
C	-1.430768	1.851979	-4.449850	-1.455386	1.863610	-4.441957
C	-1.948449	1.679368	-3.111369	-1.977674	1.691739	-3.108330
C	2.306540	0.038004	-3.464210	2.282596	0.045888	-3.447953
C	2.208489	0.476965	-4.827100	2.190807	0.483066	-4.816306
C	1.041932	1.096210	-5.349348	1.029837	1.100334	-5.329857
C	-0.071999	1.298648	-4.485549	-0.096863	1.308060	-4.466741
C	-2.244106	2.473142	-5.404674	-2.259296	2.483120	-5.407596
C	-3.542156	2.919316	-5.068871	-3.557157	2.931456	-5.086828
C	-4.010169	2.729722	-3.746105	-4.031835	2.742953	-3.766489
C	-3.226886	2.110710	-2.759202	-3.259784	2.126410	-2.770636
N	0.954837	-1.037360	2.356683	0.989528	-1.052514	2.346033
C	-0.116341	-0.831217	3.204632	-0.085029	-0.848594	3.187429
C	-1.212168	-0.228313	2.588490	-1.185930	-0.238567	2.575760
C	-2.305995	-0.037372	3.467018	-2.282350	-0.045963	3.450557
C	-2.207914	-0.476258	4.829930	-2.190549	-0.483111	4.818920
C	-1.041536	-1.095945	5.352051	-1.029564	-1.100344	5.332480
C	0.072416	-1.298336	4.488268	0.097119	-1.308113	4.469352
C	1.431035	-1.852027	4.452462	1.455651	-1.863639	4.444574
C	1.948905	-1.678973	3.114110	1.977922	-1.691808	3.110935
C	2.243939	-2.474218	5.406987	2.259668	-2.482896	5.410288
C	3.542069	-2.920207	5.071241	3.557656	-2.930928	5.089607
C	4.010313	-2.730067	3.748634	4.032316	-2.742466	3.769256
C	3.227350	-2.110298	2.761952	3.260109	-2.126293	2.773295

H	3.230842	-0.427343	-3.112073	3.202434	-0.424271	-3.090970
H	1.002330	1.420968	-6.389459	0.988262	1.424391	-6.370364
H	-1.858362	2.606600	-6.419028	-1.862499	2.612767	-6.418502
H	-5.008573	3.066300	-3.464992	-5.032348	3.080767	-3.493154
N	-3.800398	1.955150	-1.451874	-3.836017	1.966884	-1.463826
H	-3.230220	0.428169	3.114937	-3.202159	0.424270	3.093596
H	-1.002169	-1.421276	6.391991	-0.987950	-1.424306	6.373016
H	1.857560	-2.609183	6.420900	1.862841	-2.612613	6.421172
H	5.008623	-3.066871	3.467457	5.032948	-3.079994	3.496004
N	3.794143	-1.970811	1.450051	3.830990	-1.990749	1.461038
H	2.387931	-0.925812	-0.100638	2.405123	-0.931386	-0.131829
H	-2.387357	0.926511	0.103461	-2.404902	0.931249	0.134414
C	3.439040	0.241030	-5.721340	3.422529	0.242219	-5.706901
C	-4.394784	3.593911	-6.151476	-4.401926	3.605175	-6.177098
C	-3.438074	-0.239354	5.724451	-3.422296	-0.242328	5.709498
C	4.394609	-3.595007	6.153787	4.402581	-3.604276	6.179985
C	5.774503	-4.020111	5.631822	5.785587	-4.031059	5.667519
H	6.343024	-4.496839	6.445965	6.349217	-4.506701	6.485806
H	5.691628	-4.748474	4.810329	5.707100	-4.760582	4.846568
H	6.358350	-3.156833	5.276696	6.371656	-3.168493	5.314200
C	4.603038	-2.609953	7.320072	4.605160	-2.618291	7.346344
H	5.228505	-3.071885	8.101959	5.223702	-3.079397	8.134370
H	5.106841	-1.694859	6.970017	5.112896	-1.704657	6.998093
H	3.648012	-2.316601	7.781321	3.646914	-2.322222	7.799395
C	3.663365	-4.852228	6.662368	3.668892	-4.860475	6.687191
H	4.269109	-5.360890	7.430665	4.268213	-5.367257	7.461909
H	2.689374	-4.603868	7.110197	2.691269	-4.610539	7.126444
H	3.487349	-5.559729	5.836407	3.498966	-5.570124	5.861768
C	-3.232018	-0.744860	7.160052	-3.218424	-0.745472	7.146026
H	-4.141420	-0.549237	7.750644	-4.128110	-0.547941	7.735282
H	-3.042517	-1.828984	7.185705	-3.030224	-1.829828	7.173669
H	-2.393853	-0.231009	7.655298	-2.380303	-0.231433	7.641297
C	-3.736382	1.270984	5.793128	-3.718656	1.268513	5.773822
H	-4.601297	1.460583	6.451081	-4.586711	1.460699	6.426580
H	-2.868739	1.817642	6.196023	-2.852495	1.814945	6.180149
H	-3.968335	1.687562	4.801660	-3.945670	1.683783	4.780472
C	-4.652036	-0.981182	5.132045	-4.635704	-0.983694	5.115572
H	-5.536590	-0.842524	5.776417	-5.522519	-0.839714	5.755370
H	-4.906008	-0.613615	4.126719	-4.885265	-0.619620	4.107682
H	-4.445202	-2.060738	5.056139	-4.431660	-2.064135	5.045229
C	-5.774571	4.019271	-5.629434	-5.784807	4.032260	-5.664543
H	-6.343157	4.495842	-6.443623	-6.348327	4.508165	-6.482753

H	-6.358447	3.156134	-5.274013	-6.371100	3.169804	-5.311328
H	-5.691520	4.747834	-4.808137	-5.706106	4.761651	-4.843495
C	-3.663506	4.850937	-6.660491	-3.667912	4.861246	-6.684152
H	-4.269318	5.359451	-7.428832	-4.267115	5.368291	-7.458789
H	-3.487311	5.558639	-5.834741	-3.497779	5.570739	-5.858639
H	-2.689601	4.602380	-7.108397	-2.690366	4.611107	-7.123460
C	-4.603456	2.608565	-7.317471	-4.604796	2.619400	-7.343583
H	-5.228983	3.070347	-8.099400	-5.223234	3.080776	-8.131533
H	-3.648515	2.315004	-7.778764	-3.646639	2.323137	-7.796696
H	-5.107296	1.693610	-6.967108	-5.112767	1.705855	-6.995442
C	3.233104	0.746833	-7.156854	3.218669	0.745387	-7.143424
H	4.142778	0.551881	-7.747248	4.128337	0.547810	-7.732691
H	2.395360	0.232659	-7.652479	2.380512	0.231401	-7.638689
H	3.043010	1.830858	-7.182249	3.030531	1.829755	-7.171054
C	4.652414	0.983376	-5.128375	4.635987	0.983511	-5.112984
H	5.537230	0.845405	-5.772535	5.522784	0.839490	-5.752798
H	4.444946	2.062792	-5.052217	4.432003	2.063963	-5.042626
H	4.906308	0.615662	-4.123082	4.885543	0.619412	-4.105103
C	3.738226	-1.269118	-5.790370	3.718802	-1.268637	-5.771243
H	4.603433	-1.458033	-6.448134	4.586840	-1.460867	-6.424010
H	3.970137	-1.685850	-4.798957	3.945802	-1.683929	-4.777898
H	2.871007	-1.816152	-6.193666	2.852607	-1.815017	-6.177566
C	-6.088214	-1.528297	-0.621713	-6.175482	-1.499031	-0.688537
C	-5.832479	-0.588090	0.381966	-5.903547	-0.579995	0.329770
C	-5.084904	0.559940	0.116579	-5.142955	0.563465	0.082250
C	-4.579245	0.807285	-1.179518	-4.640463	0.830783	-1.210564
C	-4.830955	-0.152524	-2.183384	-4.912112	-0.105875	-2.230107
C	-5.573566	-1.301081	-1.900234	-5.667151	-1.250381	-1.965424
H	-6.675611	-2.424824	-0.408498	-6.770064	-2.393462	-0.488653
H	-6.213573	-0.751042	1.393604	-6.281252	-0.755895	1.340821
H	-4.892760	1.270520	0.921517	-4.940560	1.257295	0.899149
H	-4.445977	0.000899	-3.191275	-4.528469	0.059334	-3.236671
H	-5.754596	-2.023716	-2.700774	-5.858305	-1.956469	-2.778456
C	-4.095698	5.621881	0.671546	-4.019724	5.584272	0.759624
C	-2.856259	5.140955	0.238242	-2.797937	5.087495	0.295413
C	-2.769686	3.922669	-0.437288	-2.746759	3.886968	-0.414748
C	-3.927788	3.171562	-0.708672	-3.923368	3.167427	-0.690135
C	-5.173777	3.666221	-0.284377	-5.151630	3.679154	-0.235361
C	-5.249967	4.876705	0.409464	-5.193429	4.872173	0.491086
H	-4.161699	6.572041	1.207436	-4.056667	6.520633	1.321977
H	-1.944317	5.710492	0.437319	-1.870545	5.630774	0.495120
H	-1.796924	3.544392	-0.757453	-1.787997	3.499546	-0.763520

H	-6.082685	3.102589	-0.502329	-6.075836	3.141733	-0.455263
H	-6.225876	5.247124	0.735265	-6.157922	5.253995	0.837800
C	3.910996	-5.644476	-0.678345	3.915641	-5.642547	-0.713292
C	5.090524	-4.920633	-0.478516	5.103706	-4.941420	-0.482804
C	5.074780	-3.706750	0.213097	5.095345	-3.738810	0.228228
C	3.862754	-3.187120	0.701308	3.884877	-3.203544	0.703456
C	2.678746	-3.917050	0.491334	2.693776	-3.913164	0.468144
C	2.705760	-5.137029	-0.184084	2.712448	-5.123340	-0.226905
H	3.930197	-6.597137	-1.213402	3.927314	-6.584470	-1.267361
H	6.040925	-5.310955	-0.852496	6.053799	-5.340269	-0.848138
H	6.005506	-3.162202	0.380495	6.031691	-3.211010	0.417735
H	1.732263	-3.521607	0.864030	1.748552	-3.510532	0.835474
H	1.772619	-5.686812	-0.334203	1.773937	-5.657114	-0.397082
C	6.238119	1.405914	0.635169	6.329290	1.352341	0.650001
C	5.896861	0.510984	-0.384007	5.988511	0.452414	-0.365034
C	5.093317	-0.599313	-0.123683	5.170025	-0.646870	-0.105689
C	4.623579	-0.857416	1.183269	4.680318	-0.890207	1.196769
C	4.962878	0.056180	2.203127	5.018595	0.028686	2.212139
C	5.755919	1.171528	1.925152	5.829115	1.131887	1.935132
H	6.865372	2.275384	0.426006	6.969270	2.211994	0.439086
H	6.253467	0.679994	-1.403916	6.359498	0.608424	-1.381774
H	4.829111	-1.273457	-0.939283	4.915055	-1.326994	-0.919814
H	4.609035	-0.108548	3.220613	4.646977	-0.118579	3.225936
H	6.003379	1.860449	2.737646	6.071378	1.824430	2.746505
			tBisICz-PhCz			
C	1.372121	-0.523292	-0.071343	1.389207	-0.533188	-0.075633
C	0.765107	0.017715	-1.222888	0.756461	0.024883	-1.219502
C	-0.607025	0.556334	-1.103239	-0.655001	0.571344	-1.090122
C	-1.371671	0.523684	0.074082	-1.388993	0.533037	0.078216
C	-0.764686	-0.017399	1.225606	-0.756249	-0.025040	1.222083
C	0.607423	-0.556071	1.105942	0.655215	-0.571495	1.092704
N	-0.954467	1.037557	-2.353999	-0.989313	1.052366	-2.343450
C	0.116742	0.831485	-3.201927	0.085246	0.848451	-3.184846
C	1.212633	0.228742	-2.585740	1.186154	0.238439	-2.573172
C	-1.430768	1.851979	-4.449850	-1.455386	1.863610	-4.441957
C	-1.948449	1.679368	-3.111369	-1.977674	1.691739	-3.108330
C	2.306540	0.038004	-3.464210	2.282596	0.045888	-3.447953
C	2.208489	0.476965	-4.827100	2.190807	0.483066	-4.816306
C	1.041932	1.096210	-5.349348	1.029837	1.100334	-5.329857
C	-0.071999	1.298648	-4.485549	-0.096863	1.308060	-4.466741
C	-2.244106	2.473142	-5.404674	-2.259296	2.483120	-5.407596
C	-3.542156	2.919316	-5.068871	-3.557157	2.931456	-5.086828

C	-4.010169	2.729722	-3.746105	-4.031835	2.742953	-3.766489
C	-3.226886	2.110710	-2.759202	-3.259784	2.126410	-2.770636
N	0.954837	-1.037360	2.356683	0.989528	-1.052514	2.346033
C	-0.116341	-0.831217	3.204632	-0.085029	-0.848594	3.187429
C	-1.212168	-0.228313	2.588490	-1.185930	-0.238567	2.575760
C	-2.305995	-0.037372	3.467018	-2.282350	-0.045963	3.450557
C	-2.207914	-0.476258	4.829930	-2.190549	-0.483111	4.818920
C	-1.041536	-1.095945	5.352051	-1.029564	-1.100344	5.332480
C	0.072416	-1.298336	4.488268	0.097119	-1.308113	4.469352
C	1.431035	-1.852027	4.452462	1.455651	-1.863639	4.444574
C	1.948905	-1.678973	3.114110	1.977922	-1.691808	3.110935
C	2.243939	-2.474218	5.406987	2.259668	-2.482896	5.410288
C	3.542069	-2.920207	5.071241	3.557656	-2.930928	5.089607
C	4.010313	-2.730067	3.748634	4.032316	-2.742466	3.769256
C	3.227350	-2.110298	2.761952	3.260109	-2.126293	2.773295
H	3.230842	-0.427343	-3.112073	3.202434	-0.424271	-3.090970
H	1.002330	1.420968	-6.389459	0.988262	1.424391	-6.370364
H	-1.858362	2.606600	-6.419028	-1.862499	2.612767	-6.418502
H	-5.008573	3.066300	-3.464992	-5.032348	3.080767	-3.493154
H	-3.230220	0.428169	3.114937	-3.202159	0.424270	3.093596
H	-1.002169	-1.421276	6.391991	-0.987950	-1.424306	6.373016
H	1.857560	-2.609183	6.420900	1.862841	-2.612613	6.421172
H	5.008623	-3.066871	3.467457	5.032948	-3.079994	3.496004
H	2.387931	-0.925812	-0.100638	2.405123	-0.931386	-0.131829
H	-2.387357	0.926511	0.103461	-2.404902	0.931249	0.134414
C	3.439040	0.241030	-5.721340	3.422529	0.242219	-5.706901
C	-4.394784	3.593911	-6.151476	-4.401926	3.605175	-6.177098
C	-3.438074	-0.239354	5.724451	-3.422296	-0.242328	5.709498
C	4.394609	-3.595007	6.153787	4.402581	-3.604276	6.179985
C	5.774503	-4.020111	5.631822	5.785587	-4.031059	5.667519
H	6.343024	-4.496839	6.445965	6.349217	-4.506701	6.485806
H	5.691628	-4.748474	4.810329	5.707100	-4.760582	4.846568
H	6.358350	-3.156833	5.276696	6.371656	-3.168493	5.314200
C	4.603038	-2.609953	7.320072	4.605160	-2.618291	7.346344
H	5.228505	-3.071885	8.101959	5.223702	-3.079397	8.134370
H	5.106841	-1.694859	6.970017	5.112896	-1.704657	6.998093
H	3.648012	-2.316601	7.781321	3.646914	-2.322222	7.799395
C	3.663365	-4.852228	6.662368	3.668892	-4.860475	6.687191
H	4.269109	-5.360890	7.430665	4.268213	-5.367257	7.461909
H	2.689374	-4.603868	7.110197	2.691269	-4.610539	7.126444
H	3.487349	-5.559729	5.836407	3.498966	-5.570124	5.861768
C	-3.232018	-0.744860	7.160052	-3.218424	-0.745472	7.146026

H	-4.141420	-0.549237	7.750644	-4.128110	-0.547941	7.735282
H	-3.042517	-1.828984	7.185705	-3.030224	-1.829828	7.173669
H	-2.393853	-0.231009	7.655298	-2.380303	-0.231433	7.641297
C	-3.736382	1.270984	5.793128	-3.718656	1.268513	5.773822
H	-4.601297	1.460583	6.451081	-4.586711	1.460699	6.426580
H	-2.868739	1.817642	6.196023	-2.852495	1.814945	6.180149
H	-3.968335	1.687562	4.801660	-3.945670	1.683783	4.780472
C	-4.652036	-0.981182	5.132045	-4.635704	-0.983694	5.115572
H	-5.536590	-0.842524	5.776417	-5.522519	-0.839714	5.755370
H	-4.906008	-0.613615	4.126719	-4.885265	-0.619620	4.107682
H	-4.445202	-2.060738	5.056139	-4.431660	-2.064135	5.045229
C	-5.774571	4.019271	-5.629434	-5.784807	4.032260	-5.664543
H	-6.343157	4.495842	-6.443623	-6.348327	4.508165	-6.482753
H	-6.358447	3.156134	-5.274013	-6.371100	3.169804	-5.311328
H	-5.691520	4.747834	-4.808137	-5.706106	4.761651	-4.843495
C	-3.663506	4.850937	-6.660491	-3.667912	4.861246	-6.684152
H	-4.269318	5.359451	-7.428832	-4.267115	5.368291	-7.458789
H	-3.487311	5.558639	-5.834741	-3.497779	5.570739	-5.858639
H	-2.689601	4.602380	-7.108397	-2.690366	4.611107	-7.123460
C	-4.603456	2.608565	-7.317471	-4.604796	2.619400	-7.343583
H	-5.228983	3.070347	-8.099400	-5.223234	3.080776	-8.131533
H	-3.648515	2.315004	-7.778764	-3.646639	2.323137	-7.796696
H	-5.107296	1.693610	-6.967108	-5.112767	1.705855	-6.995442
C	3.233104	0.746833	-7.156854	3.218669	0.745387	-7.143424
H	4.142778	0.551881	-7.747248	4.128337	0.547810	-7.732691
H	2.395360	0.232659	-7.652479	2.380512	0.231401	-7.638689
H	3.043010	1.830858	-7.182249	3.030531	1.829755	-7.171054
C	4.652414	0.983376	-5.128375	4.635987	0.983511	-5.112984
H	5.537230	0.845405	-5.772535	5.522784	0.839490	-5.752798
H	4.444946	2.062792	-5.052217	4.432003	2.063963	-5.042626
H	4.906308	0.615662	-4.123082	4.885543	0.619412	-4.105103
C	3.738226	-1.269118	-5.790370	3.718802	-1.268637	-5.771243
H	4.603433	-1.458033	-6.448134	4.586840	-1.460867	-6.424010
H	3.970137	-1.685850	-4.798957	3.945802	-1.683929	-4.777898
H	2.871007	-1.816152	-6.193666	2.852607	-1.815017	-6.177566
C	-4.624414	0.931895	-1.004529	-4.640519	0.940523	-1.005525
C	-3.885493	2.048733	-1.413864	-3.921441	2.065935	-1.426254
C	-3.923821	3.225781	-0.629753	-3.976561	3.248193	-0.650658
C	-4.673689	3.315242	0.541545	-4.728122	3.336804	0.519546
H	-4.627427	0.024582	-1.614163	-4.630055	0.028239	-1.607483
H	-3.360585	4.099695	-0.965540	-3.425712	4.126643	-0.995036
H	-4.699989	4.246502	1.108225	-4.768387	4.271561	1.079707

C	-5.404256	2.189711	0.942919	-5.440988	2.203220	0.929509
C	-5.377421	0.991662	0.177644	-5.394073	0.998368	0.176416
N	-6.265334	2.021723	2.032679	-6.303860	2.033734	2.017436
C	-6.253313	0.050137	0.847911	-6.258279	0.050718	0.853655
C	-6.786273	0.720739	1.982693	-6.807625	0.726509	1.977629
C	-7.653707	0.067797	2.869622	-7.676960	0.074845	2.863175
C	-7.992697	-1.258206	2.595131	-7.997902	-1.257847	2.600838
C	-7.481752	-1.930357	1.468578	-7.467029	-1.937386	1.487957
C	-6.609071	-1.283208	0.595392	-6.594873	-1.290059	0.614341
H	-8.043796	0.569593	3.756084	-8.082884	0.583947	3.738305
H	-8.664856	-1.786296	3.276784	-8.672055	-1.785215	3.281063
H	-7.765466	-2.969466	1.284515	-7.736189	-2.982180	1.315128
H	-6.199983	-1.808810	-0.271242	-6.172150	-1.821385	-0.242348
C	-7.299651	5.047582	4.806881	-7.400075	5.062548	4.761642
C	-8.298741	4.313804	4.159568	-8.384584	4.327513	4.093528
C	-7.963358	3.318955	3.237574	-8.028086	3.332910	3.179646
C	-6.614823	3.032783	2.968935	-6.673538	3.047914	2.942211
C	-5.613271	3.766885	3.624730	-5.686521	3.783139	3.618038
C	-5.957108	4.772338	4.531556	-6.051159	4.790321	4.514636
H	-7.565556	5.829142	5.523051	-7.682964	5.844882	5.470223
H	-9.351654	4.525440	4.363184	-9.442238	4.537543	4.273675
H	-8.746661	2.768436	2.713364	-8.798530	2.780239	2.638456
H	-4.564108	3.540035	3.430822	-4.632913	3.557818	3.445608
H	-5.165960	5.333970	5.035127	-5.272297	5.354877	5.033962
C	3.806516	-2.923724	0.434250	3.842775	-2.920493	0.437884
C	3.922336	-1.958411	1.442066	3.961099	-1.966763	1.456274
C	4.869969	-0.918532	1.294919	4.908841	-0.925265	1.316647
C	5.698056	-0.819411	0.178022	5.737499	-0.816830	0.201121
H	3.098156	-3.748474	0.545788	3.133626	-3.745414	0.542105
H	4.968795	-0.180406	2.094380	5.007442	-0.194663	2.122964
H	6.435245	-0.018834	0.109398	6.475142	-0.016030	0.138318
C	5.569453	-1.788614	-0.824961	5.606977	-1.776512	-0.810525
C	4.618837	-2.839131	-0.706478	4.654606	-2.826250	-0.702557
N	6.295191	-1.954959	-2.009449	6.335020	-1.934135	-1.994738
C	4.767242	-3.669881	-1.885876	4.804949	-3.647779	-1.888398
C	5.812262	-3.098371	-2.662212	5.854872	-3.072923	-2.655949
C	6.185703	-3.651954	-3.894800	6.239137	-3.622529	-3.886794
C	5.510006	-4.793268	-4.330053	5.565348	-4.760868	-4.332177
C	4.481173	-5.375869	-3.565990	4.528160	-5.344123	-3.579691
C	4.102961	-4.816135	-2.346795	4.141899	-4.790502	-2.360181
H	6.969236	-3.202056	-4.506272	7.031144	-3.173348	-4.487546
H	5.783607	-5.237861	-5.290597	5.848171	-5.203611	-5.290893

H	3.970551	-6.267736	-3.937888	4.019447	-6.232942	-3.960689
H	3.294238	-5.258500	-1.759702	3.328451	-5.235495	-1.781335
C	9.560094	0.450723	-3.224441	9.608695	0.470884	-3.180356
C	9.687193	-0.941411	-3.183849	9.739945	-0.920367	-3.126196
C	8.615519	-1.740073	-2.776206	8.664628	-1.718287	-2.728282
C	7.390727	-1.149460	-2.423415	7.433338	-1.127931	-2.399823
C	7.263632	0.248437	-2.470157	7.301315	0.268654	-2.461712
C	8.345838	1.040750	-2.860934	8.387404	1.061065	-2.841099
H	10.402367	1.072826	-3.537979	10.455225	1.092061	-3.483647
H	10.634502	-1.412249	-3.459309	10.692572	-1.391313	-3.382557
H	8.728070	-2.824377	-2.718634	8.778656	-2.802010	-2.659330
H	6.311237	0.712527	-2.209872	6.343354	0.731965	-2.220038
H	8.231061	2.127314	-2.894032	8.270148	2.146964	-2.884719

2.3. Photophysical characterization

2.2.1. ultraviolet-visible spectroscopy and photoluminescence analysis

For photophysical analysis, the ultraviolet-visible (UV-Vis) absorption spectra and photoluminescence (PL) spectra were obtained by UV-Vis spectrophotometer (JASCO, V-730) and fluorescence spectrophotometer (PerkinElmer, LS-55), respectively. For UV-Vis and PL measurements, the materials were sampled with THF at a concentration of 1.0×10^{-5} M. The fluorescence and phosphorescence emissions were recorded without and with 1 ms delay at 77K. The solvent dependent PL spectra were measured at room temperature.

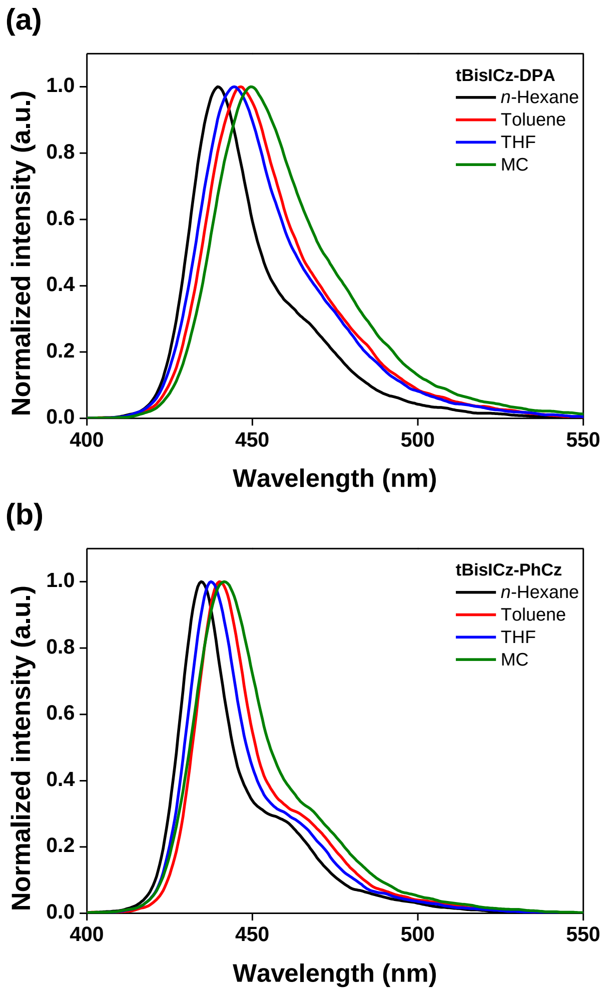

Figure S11. Solvent dependent PL spectra of (a) tBisICz-DPA and (b) tBisICz-PhCz in different solvent.

2.2.2. Transient photoluminescence and absolute photoluminescence quantum yield

In the case of absolute PL quantum yield (PLQY) and transient PL analysis, Quantaurus-QY system (Hamamatsu, C11347-11) and Quantaurus-Tau system (Hamamatsu, C11367-31) were used, and the excitation wavelengths were 290 nm and 280 nm, respectively. The measurements were carried out under a nitrogen condition using a vacuum deposited film. The internal LED / Xe lamp excitation source was used to measure prompt / delayed decay component, respectively. For P-N mixed host system, 1,3-bis(*N*-carbazolyl)benzene (mCP) and diphenyl[4-(triphenylsilyl)phenyl]phosphine oxide (TSPO1) were chosen. The thickness of the films was 40 nm, and the host materials were deposited at a ratio of 50:50 based on weight. The doping concentration of the emitters was 3 wt%. The photophysical parameters related to electronic transitions were calculated following formulas[S12]:

$$\tau_p = \frac{1}{k_p}$$

$$\tau_d = \frac{1}{k_d}$$

$$k_{ISC} = \left(1 - \phi_p\right) \cdot k_p$$

$$k_{RISC} = \frac{k_p k_d}{k_{ISC}} \cdot \frac{\phi_d}{\phi_p}$$

$$k_{nr}^{T} = k_d - \phi_p \cdot k_{RISC}$$

In which, Φ_p means prompt fluorescence PLQY, Φ_d means delayed fluorescence PLQY, τ_p means prompt decay times, k_p means rate constant of prompt fluorescence, τ_d means delayed decay times, k_d means rate constant of delayed fluorescence, k_{ISC} means rate constant of intersystem crossing (ISC), k_{RISC} means rate constant of reverse intersystem crossing (RISC), k^S_r means rate constant of radiative decay for singlet state, k^T_{nr} means rate constant of non-radiative decay for triplet state.

The parameters were calculated by setting the non-radiative rate constant of singlet state to zero.

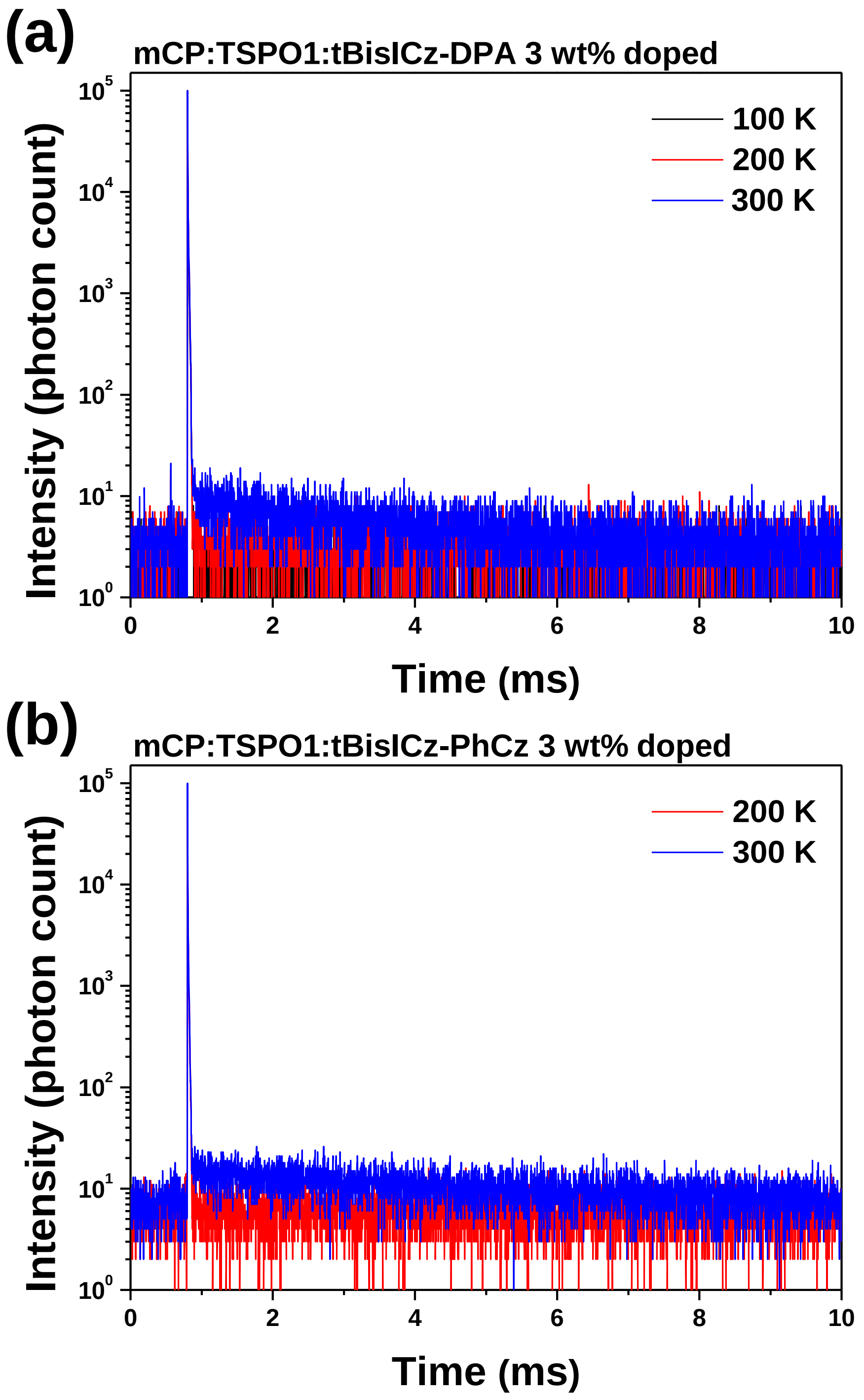

Figure S12. Temperature-dependent transient PL decay curves of (a) tBisICz-DPA and (b) tBisICz-PhCz doped mCP:TSPO1 films at 3 wt% doping concentration.

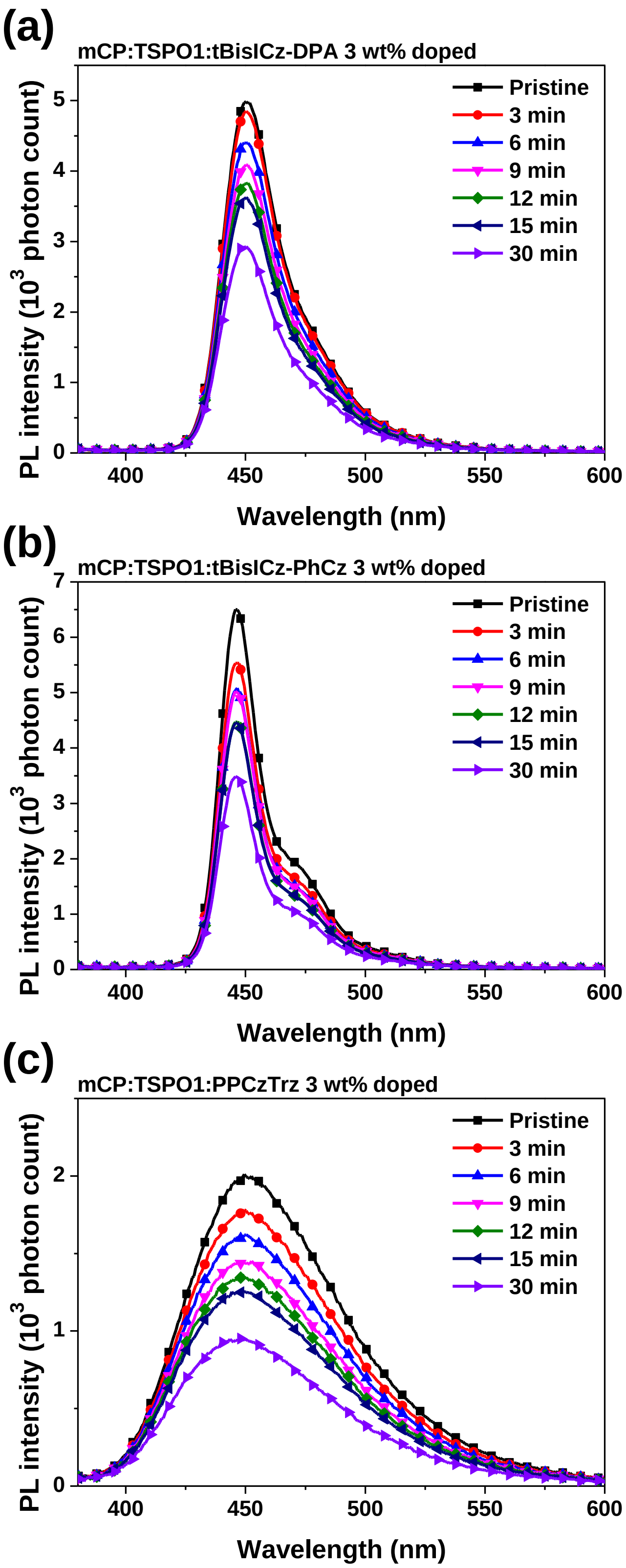

Figure S13. Solid PL spectra of (a) tBisICz-DPA, (a) tBisICz-PhCz and (a) PPCzTrz according to UV irradiation time.

2.2.3. Angle-dependent photoluminescence

Angle-dependent PL equipment consisted of a rotational stage and a half-cylinder lens (fused silica) with a refractive index matching liquid. As an excitation source, a 325 nm He/Cd laser (Kimmon Koha Co., Ltd.) was used to excite a deposited film which was attached to a half-cylinder lens. A linear p-polarizer and spectrometer (Maya 2000 Pro, Ocean Optics Inc.) were used to detect the p-polarized PL signal of the photo-excited film according to the rotational angle of the rotational stage. In this case, an ND filter was used to exclude the excitation wavelength.

2.4. Electrochemical and thermal analysis

To get electrochemical properties, the oxidation scan was obtained by cyclic voltammetry (Ivium Tech., Iviumstat) measurement to approximate the energy levels of the highest occupied molecular orbital (HOMO). This measurement was performed in an MC solution in which tetrabutylammonium perchlorate and samples were dissolved together at a concentration of 0.1 M and 1.0×10^{-3} M, respectively. For 3 electrode system, platinum wires were used as working and counter electrode with saturated Ag/AgCl reference electrode. The scan rate was 0.1 V/s. Ferrocene was used as reference. The lowest occupied molecular orbital (LUMO) was calculated from HOMO level and HOMO-LUMO gap obtained from the onset energy of UV-vis absorption spectrum. Thermogravimetric analysis (TGA, SEICO Inst., TG/DTA7300) was conducted to specify thermal properties. TGA was performed at a heating rate of 40 °C/min from room temperature to 600 °C under nitrogen to detect the decomposition temperature (T_d) at 5% weight loss.

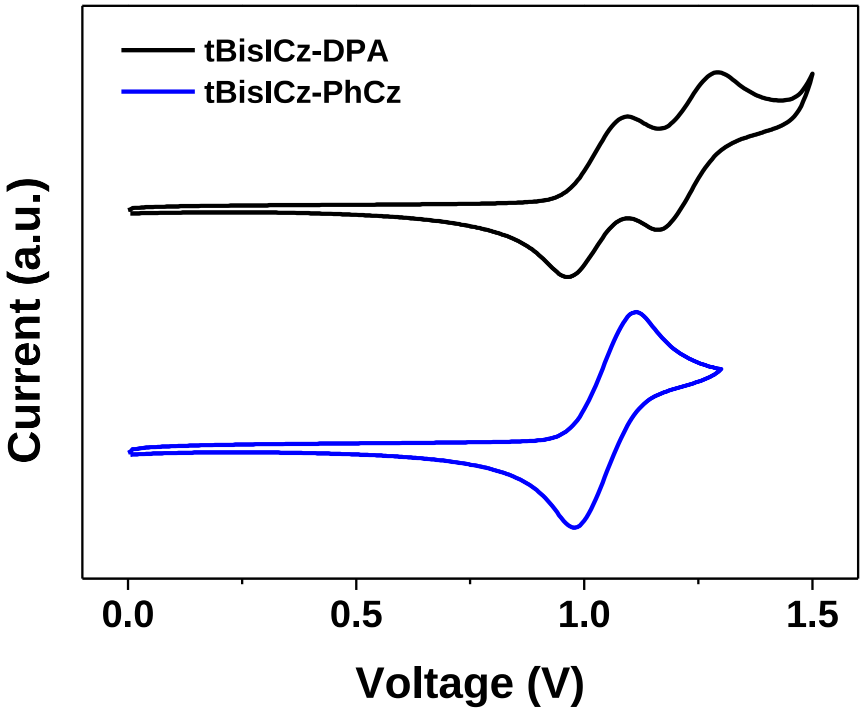

Figure S14. The oxidation curves of tBisICz-DPA (black) and tBisICz-PhCz (blue) scanned from cyclic voltammetry.

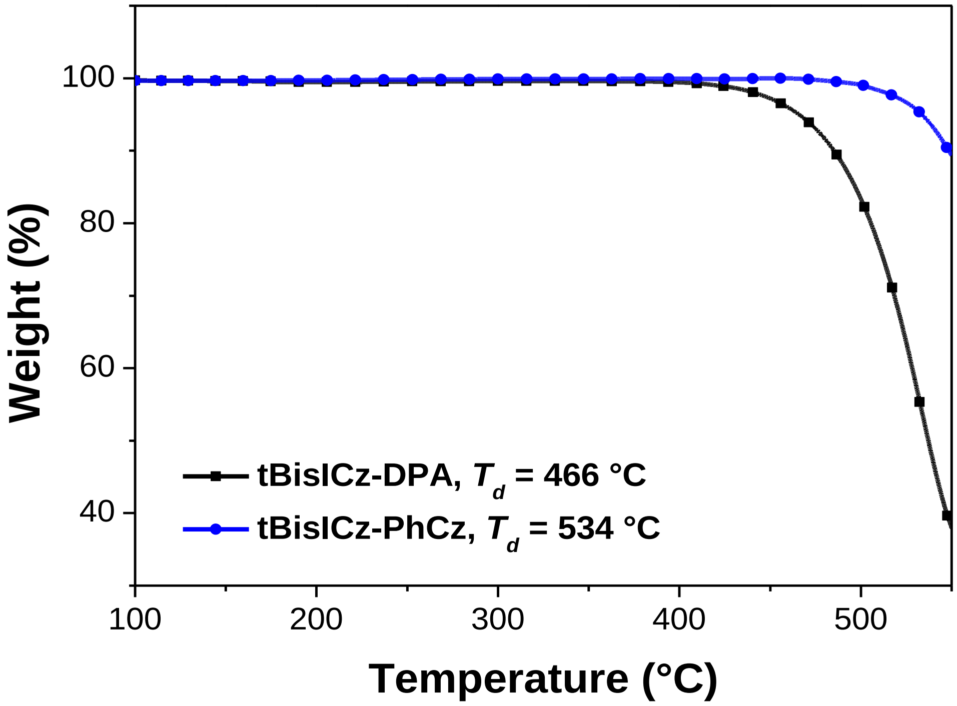

Figure S15. Thermogravimetric analysis curves. The decomposition temperature (T_d) with 5% weight loss of tBisICz-DPA (black) and tBisICz-PhCz (blue).

2.5. Device analysis

The fabricated OLEDs were prepared using a transparent glass substrate with a 50 nm thick indium tin oxide (ITO) which worked as an anode. Before fabrication process, the ITO substrates were sequentially cleaned with acetone and DW by ultrasonication. The deep blue OLED devices architecture is: PEDOT:PSS (40 nm) / TAPC (5 nm) / TCTA (5 nm) / PCZAC (5 nm) / mCP (5 nm) / mCP:TSPO1:tBisICz-DPA or tBisICz-PhCz (25 nm:50 wt%:1, 3, 5 wt%) / TSPO1 (25 nm) / LiF (1.5 nm)/Al(200 nm). In which, PEDOT:PSS stands for [poly(3,4-ethylenedioxythiophene)-poly(styrenesulfonate)] which was worked as a hole injection layer. TAPC is [(1,1-bis(4-di-*p*-tolylaminophenyl)cyclohexane)] and PCZAC is [9,9-dimethyl-10-(9-phenyl-9*H*-carbazol-3-yl)-9,10-dihydroacridine] which worked as hole transport layers. mCP stands for 1,3-di(9*H*-carbazol-9-yl)benzene which worked as an electron blocking layer and P-type host in emitting layer. TSPO1 is diphenyl[4-(triphenylsilyl)phenyl]phosphine oxide which worked as a hole blocking layer and N-type host in emitting layer.

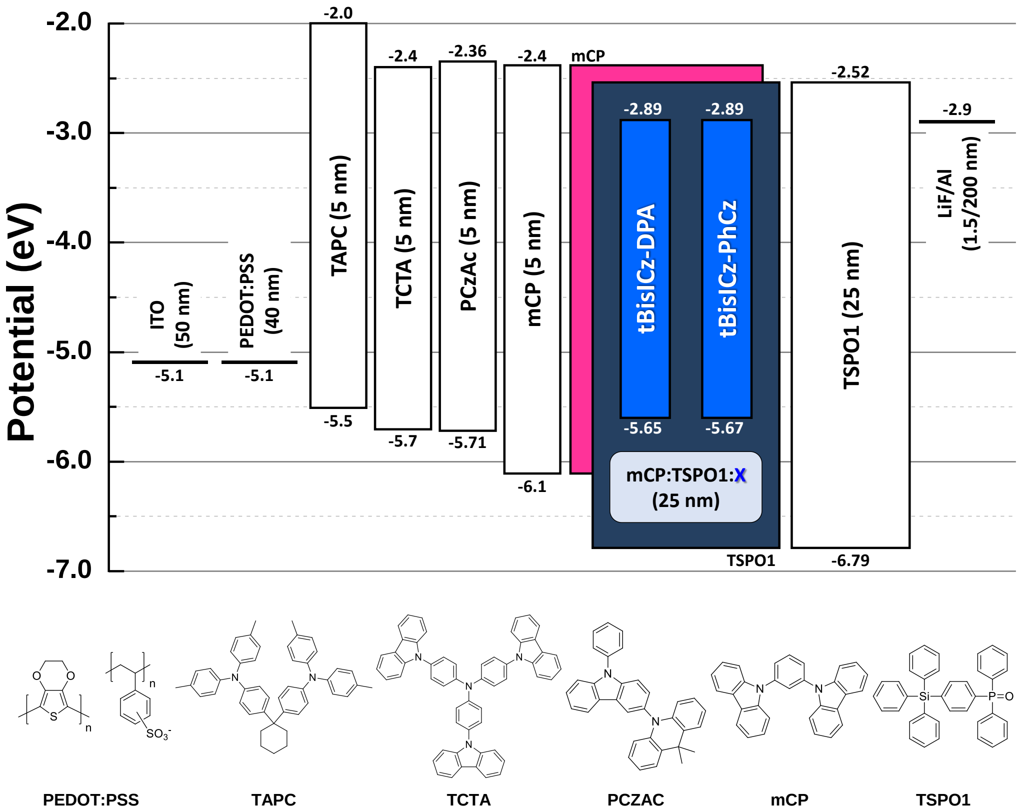

Figure S16. Device structure and chemical structure of materials used.

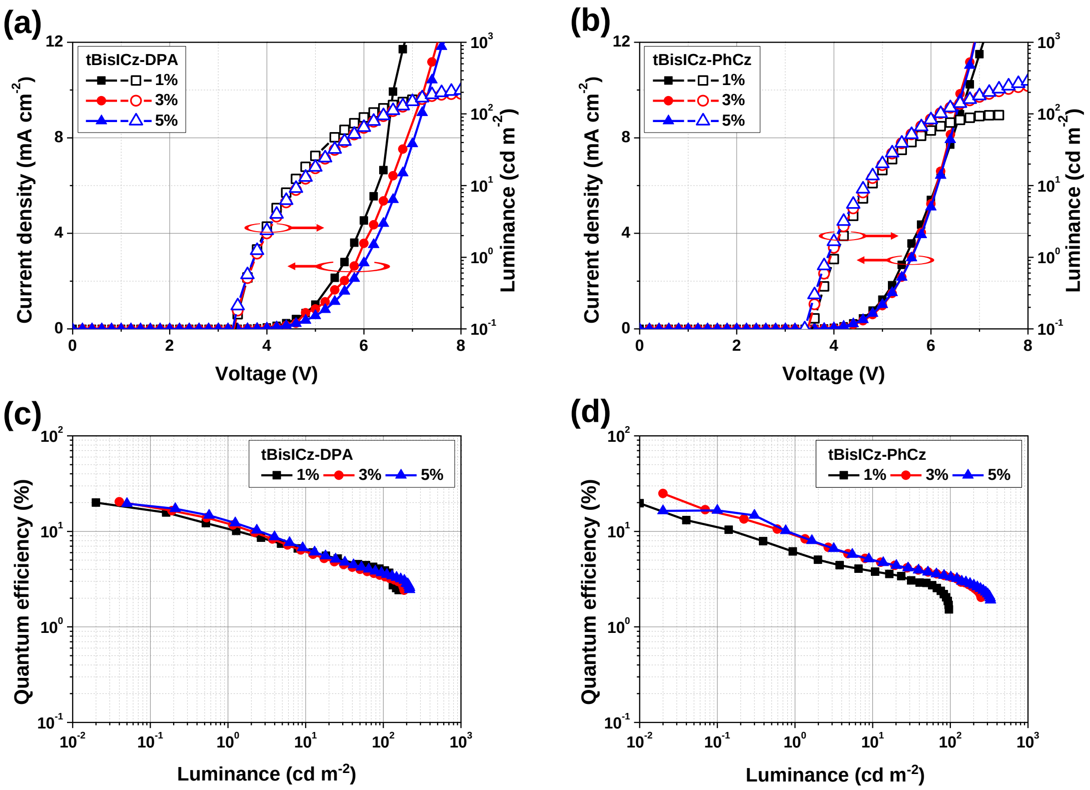

Figure S17. Current density–voltage–luminance curves of (a) tBisICz-DPA and (b) tBisICz-PhCz devices and external quantum efficiency–luminance curves of (c) tBisICz-DPA and (d) tBisICz-PhCz at different doping concentrations.

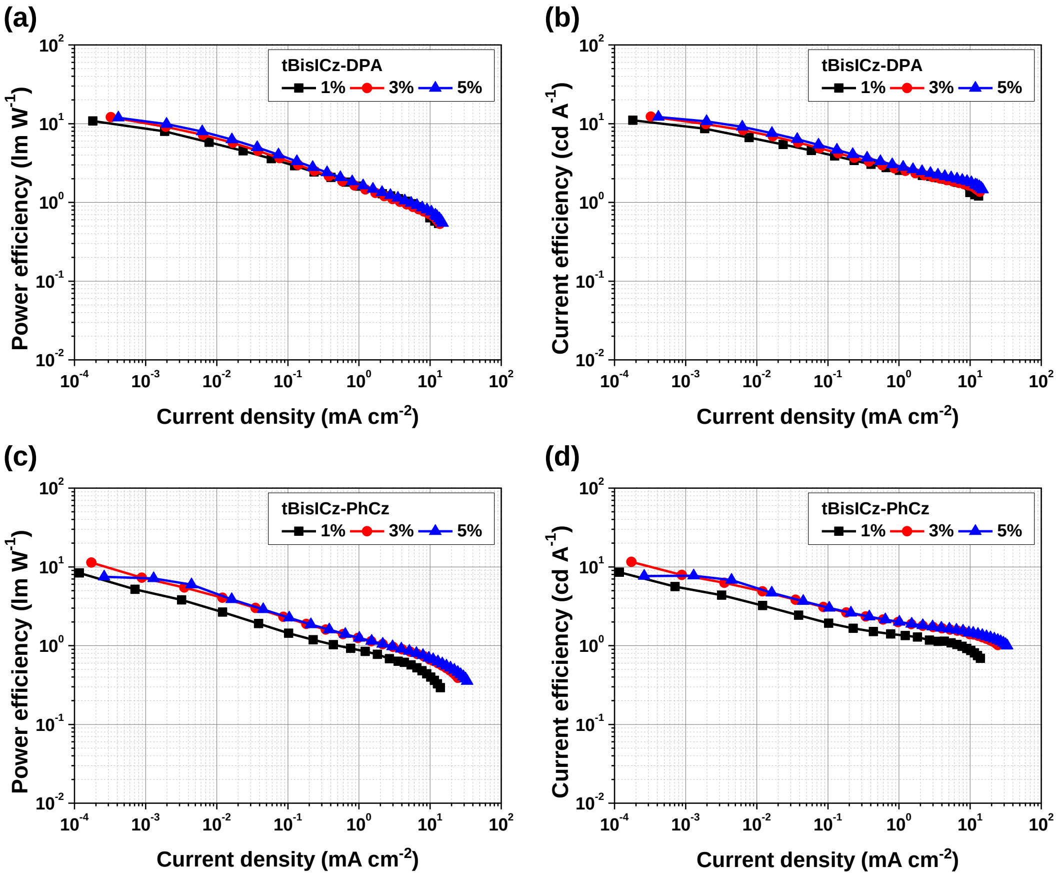

Figure S18. Power efficiency–current density curves of (a) tBisICz-DPA, (c) tBisICz-PhCz and Current efficiency–current density curves of (b) tBisICz-DPA, (d) tBisICz-PhCz at different doping concentration.

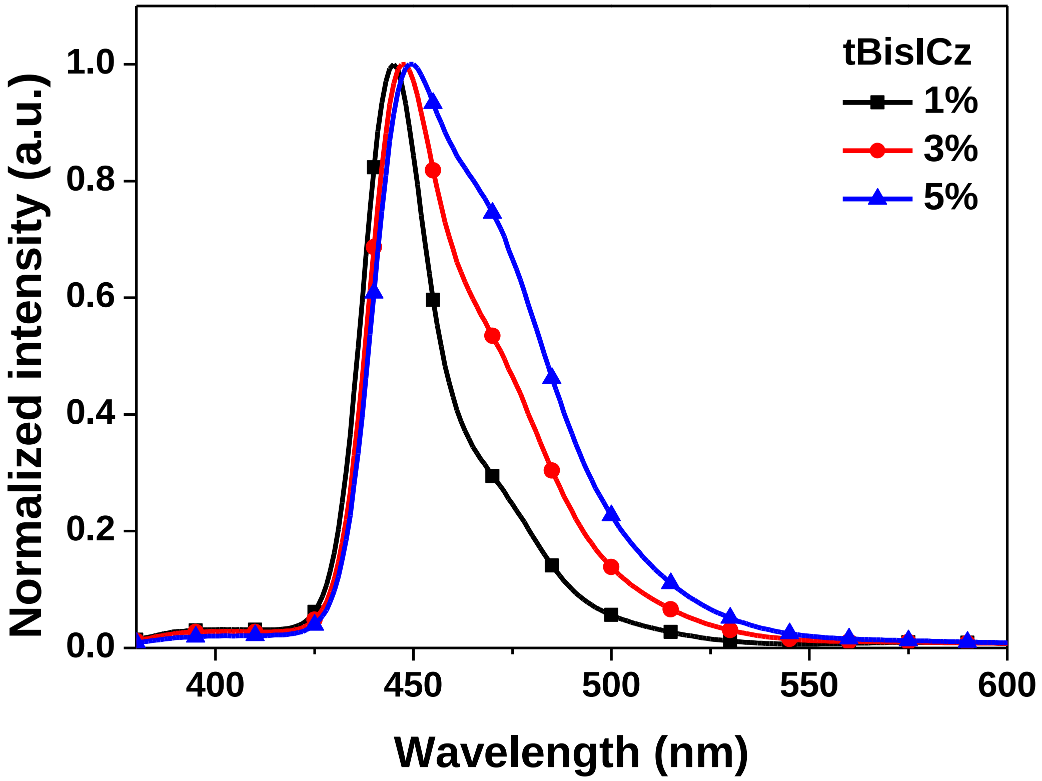

Figure S19. Normalized electroluminescence spectra of tBisICz devices at different doping concentrations.

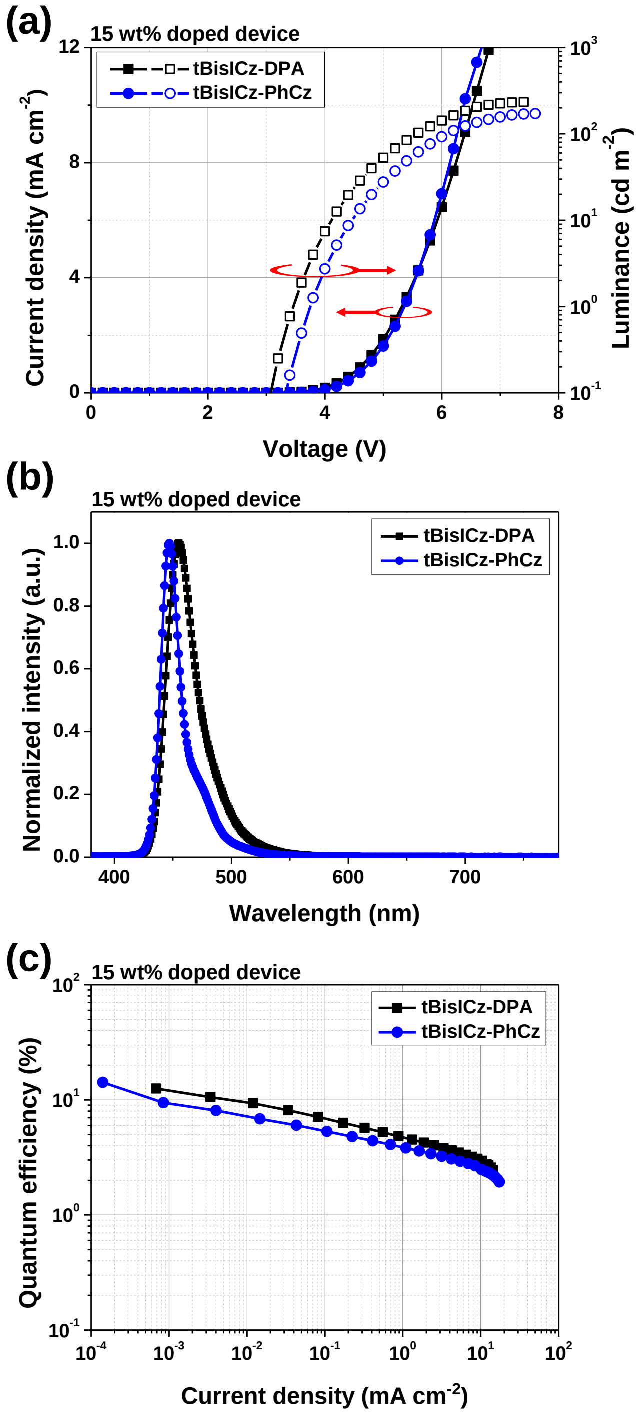

Figure S20. Device performance of 15 wt% emitter doped MR-TADF OLEDs. (a) Current density–voltage–luminance curves, (b) Normalized EL spectra, (c) External quantum efficiency–current denisty curves of tBisICz-DPA (black line) and tBisICz-PhCz (blue line) device.

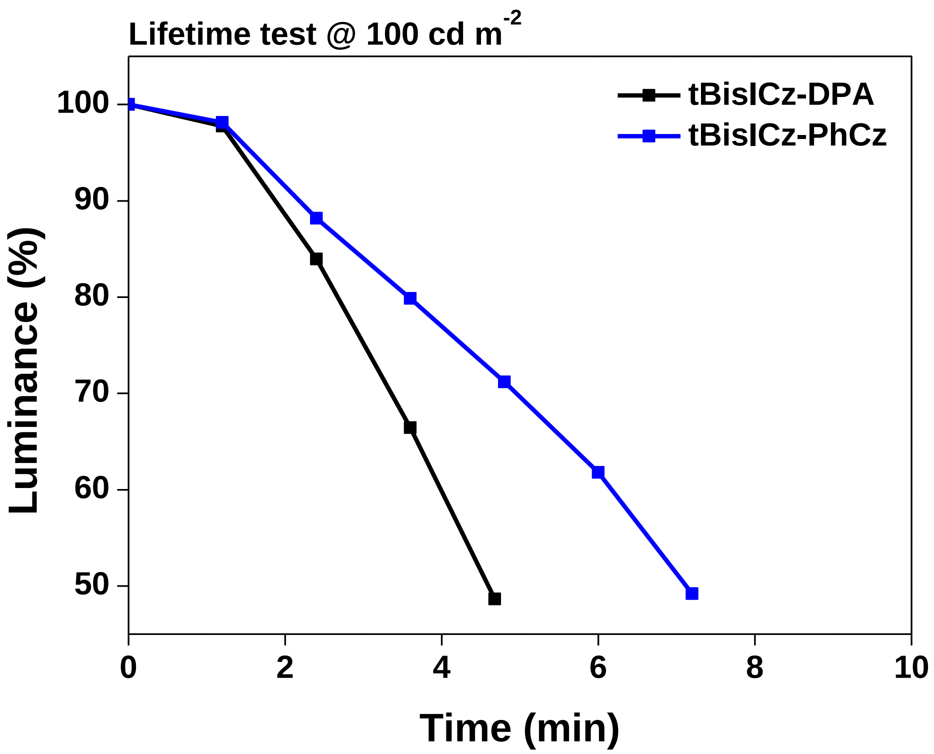

Figure S21. Device lifetime of 3 wt% doped MR-TADF OLEDs.

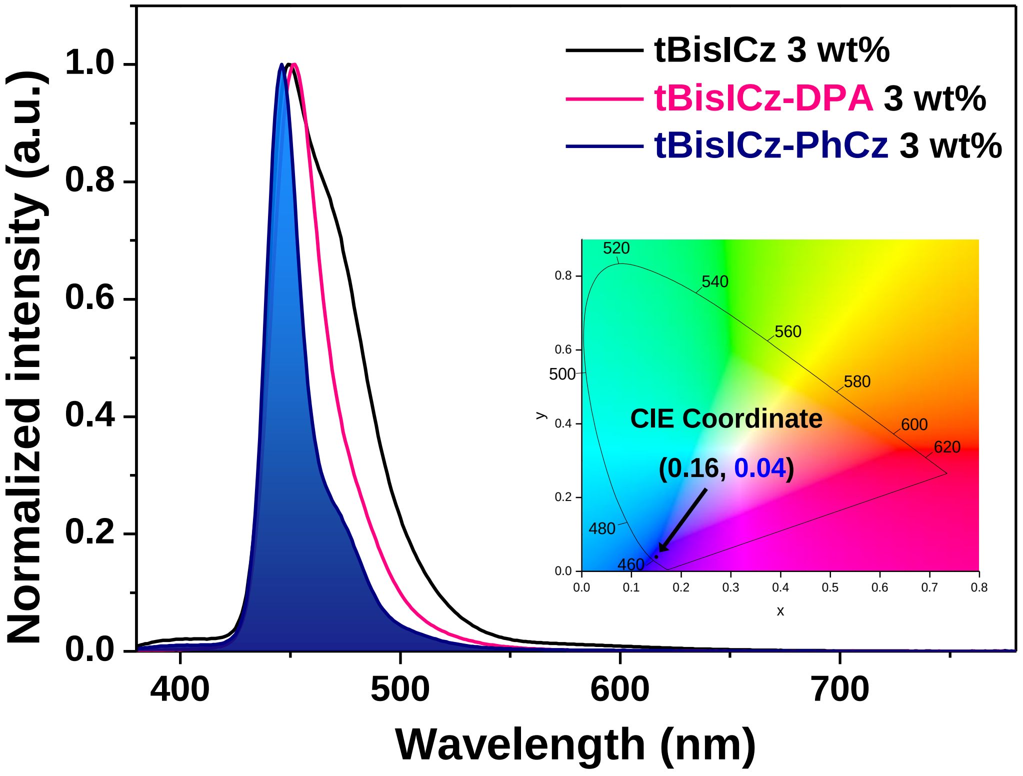

Figure S22. The electroluminescence spectra of the blue OLEDs with different emitters at 3 wt% doping concentration along with CIE color coordinate of tBisICz-PhCz device.

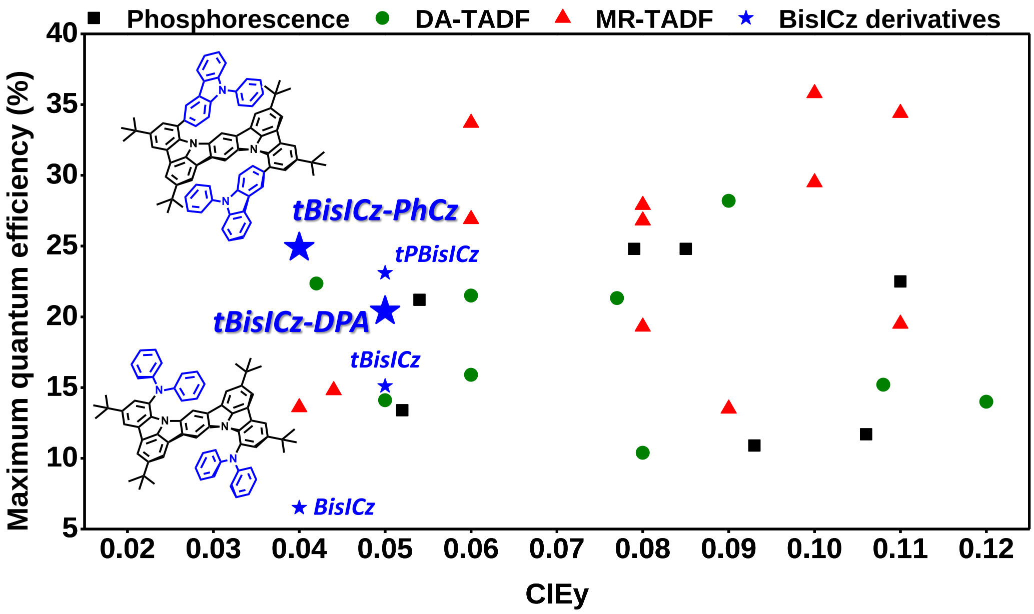

Figure S23. EQE_{Max}-CIE_y plot of deep blue OLEDs of this work and previously reported phosphorescence (black), conventional donor-acceptor type TADF (DA-TADF) (green), MR-TADF (red) and BisICz emitters (blue).

3. Supplementary table

Table S2. Calculated reverse intersystem crossing rate and its related properties.

	k_{RISC} (s^{-1})^{a}	k_{RISC} component^{b}				SOC (cm^{-1})^{e}	
		DSO	SV	ΔE_{ST} (eV)^{c}	ΔE_{TT} (eV)^{d}	S_1-T_1	S_1-T_2
**tBisICz**^{f}	150	3%	97%	0.261	0.287	0.144	0.578
**tBisICz-DPA**	168	4%	96%	0.243	0.283	0.164	0.856
**tBisICz-PhCz**	41	5%	95%	0.262	0.311	0.130	0.829

^{a} Determined from the analytical solution of the Fermi's golden rule expression. ^{b} Direct spin-orbit and spin–vibronic contributions to total k_{RISC}. ^{c} Adiabatic singlet-triplet energy difference at the SCS-ADC(2) level. ^{d} Vertical triplet-triplet energy differences calculated at the ω*B97M-V level using the S_1 geometry. The optimized ω values are 0.120 for tBisICz-DPA and 0.118 for tBisICz-PhCz. ^{e} Triplet substate-averaged spin–orbit coupling. ^{f} Data are taken from reference [15].

Table S3. Summarized device performance data of 15 wt% doped MR-TADF OLEDs.

Emitter	Doping conc.	V_d^{a} (V)	λ_{EL}^{a} (nm)	FWHM^{a} (nm)	CIE (x, y)^{a}	EQE_{Max} (%)	PE_{Max} (lm W^{-1})	CE_{Max} (Cd A^{-1})
**tBisICz-DPA**	15 wt%	5.6	455	30	(0.14, 0.07)	12.5	9.2	8.8
**tBisICz-PhCz**	15 wt%	4.5	447	19	(0.15, 0.04)	14.2	7.4	7.1

^{a} Values at 100 cd m^{-2}.

Table S4. Summary of device performances of deep blue OLEDs reported in other works compared with those of this work.

Emitter	Emitter type	λ_{EL}^{a} (nm)	$FWHM_{EL}$^{a} (nm)	EQE_{Max}^{b} (%)	CIE (x, y)	Ref
**Ir(fbppz)$_2$(dfbdp)**	Ph.^{c}	455	-	11.7	(0.155, 0.106)	S13
**Ir(fdpt)$_3$**		431, 458	-	22.5	(0.15, 0.11)	S14
**PtON6-tBu**		452	30	10.9	(0.147, 0.093)	S15
**PtON7-dtb**		451	29	24.8	(0.148, 0.079)	
***mer*-Ir1**		445	-	24.8	(0.149, 0.085)	S16
***mer*-Ir(CF$_3$pbp)$_3$**		407, 423	-	21.2	(0.161, 0.054)	S17
**2**		430	63.1	13.4	(0.154, 0.052)	S18
**CzBPCN**	DA-TADF^{d}	460	48	14	(0.14, 0.12)	S19
**TDBA-Ac**		445	48	21.5	(0.15, 0.06)	S20
**DMAC2PTO**		448	52	15.2	(0.154, 0.108)	S21
**TDBA-SAF**		456	55	28.2	(0.142, 0.090)	S22
**TDBA-PAS**		435	50	22.35	(0.155, 0.042)	S23
**TDBA-DPAC**		449	59	21.32	(0.150, 0.077)	
**TB-tCz**		412	44.2	15.9	(0.17, 0.06)	S24
**TB-tPCz**		420	43.5	14.1	(0.17, 0.05)	
**BO3N**		450	55	10.39	(0.14, 0.08)	S25
**DABNA-1**	MR-TADF^{e}	459	28	13.5	(0.13, 0.09)	5
**DABNA-TP-TB**		457	33	19.5	(0.14, 0.11)	10
**v-DABNA**		469	18	34.4	(0.12, 0.11)	8

**v-DABNA-O-Me**		465	23	29.5	(0.13, 0.10)	9
**2B-DTACrs**		447	26	14.8	(0.150, 0.044)	S26
**4F-v-DABNA**		464	18	35.8	(0.13, 0.10)	S27
**4F-m-v-DABNA**		461	18	33.7	(0.13, 0.06)	
**BOBO-Z**		445	18	13.6	(0.15, 0.04)	
**BOBS-Z**		456	23	26.9	(0.14, 0.06)	S28
**BSBS-Z**		463	22	26.8	(0.13, 0.08)	
**t-DAB-DPA**		459	26	27.9	(0.13, 0.08)	S29
**3tPAB**		460	26	19.3	(0.14, 0.08)	S30
**BisICz**		437	24	6.5	(0.16, 0.04)	
**tBisICz**		445	22	15.1	(0.16, 0.05)	15
**tPBisICz**	BisICz derivatives[f]	452	21	23.1	(0.15, 0.05)	
**tBisICz-DPA**		452	28	20.4	(0.15, 0.05)	**This work**
**tBisICz-PhCz**		446	19	24.9	(0.16, 0.04)	

[a] Full-width-at-half-maximum of electroluminescence spectrum. [b] Maximum external quantum efficiency. [c] Ph. represents phosphorescence. [d] DA-TADF represents conventional donor-acceptor type thermally activated delayed fluorescence (TADF). [e] MR-TADF represents multi-resonance TADF. [f] BisICz is material name of indolo[3,2,1-*jk*]indolo[1′,2′,3′:1,7]indolo[3,2-*b*]carbazole which was reported in reference 15.

Supplementary Reference

[S1] V. C. Gibson, S. K. Spitzmesser, A. J. White, D. J. Williams, *Dalton Trans.* **2003**, *46*, 2718.

[S2] S. G. Balasubramani, G. P. Chen, S. Coriani, M. Diedenhofen, M. S. Frank, Y. J. Franzke, F. Furche, R. Grotjahn, M. E. Harding, C. Hättig, *J. chem. phys.* **2020**, *152*, 184107.

[S3] A. Becke, *J. Chem. Phys.* **1993**, *98*: 5648.

[S4] L. Chengteh, Y. Weitao, G. P. Robert, *Phys. Rev. B* **1988**, *37*, 785.

[S5] S. H. Vosko, L. Wilk, M. Nusair, *Can. J. Phys.* **1980**, *58*, 1200.

[S6] P. J. Stephens, F. J. Devlin, C. F. Chabalowski, M. J. Frisch, *J. Phys. Chem.* **1994**, *98*, 11623.

[S7] S. Hirata, M. Head-Gordon, *Chem. Phys. Lett.* **1999**, *314*, 291.

[S8] M. Gerenkamp, S. Grimme, *Chem. Phys. Lett.* **2004**, *392*, 229.

[S9] J. Schirmer, *Phys. Rev. A* **1982**, *26*, 2395.

[S10] N. Mardirossian, M. Head-Gordon, *J. Chem. Phys.* **2016**, *144*, 214110.

[S11] E. Epifanovsky, A. T. Gilbert, X. Feng, J. Lee, Y. Mao, N. Mardirossian, P. Pokhilko, A. F. White, M. P. Coons, A. L. Dempwolff, *J. Chem. Phys.* **2021**, *155*, 084801.

[S12] K. Masui, H. Nakanotani, C. Adachi, *Org. Electron.* **2013**, *14*, 2721.

[S13] Y. C. Chiu, J. Y. Hung, Y. Chi, C. C. Chen, C. H. Chang, C. C. Wu, Y. M. Cheng, Y. C. Yu, G. H. Lee, P. T. Chou, *Adv. Mater.* **2009**, *21*, 2221.

[S14] X. Li, J. Zhang, Z. Zhao, L. Wang, H. Yang, Q. Chang, N. Jiang, Z. Liu, Z. Bian, W. Liu, *Adv. Mater.* **2018**, *30*, 1705005.

[S15] T. Fleetham, G. Li, L. Wen, J. Li, *Adv. Mater.* **2014**, *26*, 7116.

[S16] H. Y. Park, A. Maheshwaran, C. K. Moon, H. Lee, S. S. Reddy, V. G. Sree, J. Yoon, J. W. Kim, J. H. Kwon, J. J. Kim, *Adv. Mater.* **2020**, *32*, 2002120.
[S17] R. Kumaresan, H. Y. Park, A. Maheshwaran, H. Park, Y. Do, M. Song, J. Yoon, S. I. Ahn, S. H. Jin, *Adv. Opt. Mater.* **2022**, *10*, 2101686.
[S18] A. K. Pal, S. Krotkus, M. Fontani, C. F. Mackenzie, D. B. Cordes, A. M. Slawin, I. D. Samuel, E. Zysman-Colman, *Adv. Mater.* **2018**, *30*, 1804231.
[S19] Y. J. Cho, S. K. Jeon, S.-S. Lee, E. Yu, J. Y. Lee, *Chem. Mater.* **2016**, *28*, 5400.
[S20] D. H. Ahn, S. W. Kim, H. Lee, I. J. Ko, D. Karthik, J. Y. Lee, J. H. Kwon, *Nat. Photonics* **2019**, *13*, 540.
[S21] S. Sun, R. Guo, Q. Zhang, X. Lv, P. Leng, Y. Wang, Z. Huang, L. Wang, *Dyes Pigm.* **2020**, *178*, 108367.
[S22] H. Lim, H. J. Cheon, S. J. Woo, S. K. Kwon, Y. H. Kim, J. J. Kim, *Adv. Mater.* **2020**, *32*, 2004083.
[S23] H. J. Tan, G. X. Yang, Y. L. Deng, C. Cao, J. H. Tan, Z. L. Zhu, W. C. Chen, Y. Xiong, J. X. Jian, C. S. Lee, *Adv. Mater.* **2022**, *34*, 2200537.
[S24] H. J. Kim, H. Kang, J. E. Jeong, S. H. Park, C. W. Koh, C. W. Kim, H. Y. Woo, M. J. Cho, S. Park, D. H. Choi, *Adv. Funct. Mater.* **2021**, *31*, 2102588.
[S25] Y. Gan, X. Peng, W. Qiu, L. Wang, D. Li, W. Xie, D. Liu, M. Li, J. Lin, S.-J. Su, *Chem. Eng. J.* **2022**, *430*, 133030.
[S26] C.-Y. Chan, S. M. Suresh, Y.-T. Lee, Y. Tsuchiya, T. Matulaitis, D. Hall, A. M. Slawin, S. Warriner, D. Beljonne, Y. Olivier, *Chem. Commum.* **2022**, *58*, 9377.
[S27] K. R. Naveen, H. Lee, R. Braveenth, K. J. Yang, S. J. Hwang, J. H. Kwon, *Chem. Eng. J.* **2022**, *432*, 134381.
[S28] I. S. Park, M. Yang, H. Shibata, N. Amanokura, T. Yasuda, *Adv. Mater.* **2022**, *34*, 2107951.
[S29] J. Kim, W. Chung, J. Kim, J. Lee, *Mater. Today Energy* **2021**, *21*, 100792.
[S30] Y. Wang, Y. Duan, R. Guo, S. Ye, K. Di, W. Zhang, S. Zhuang, L. Wang, *Org. Electron.* **2021**, *97*, 106275.
